# Miniaturized IL-2/anti-IL-2 immunocytokines selectively activate and support the *in vivo* persistence of regulatory T cells

**DOI:** 10.3389/fimmu.2026.1755812

**Published:** 2026-03-09

**Authors:** Charina S. Fabilane, Jakub Tomala, Paul M. Zdinak, Bailey T. Chalmers, A. Carson Stephenson, Emily Ariail, Ella C. Teeley, Alok V. Joglekar, Jamie B. Spangler

**Affiliations:** 1 Program in Molecular Biophysics, Johns Hopkins University, Baltimore, MD, United States; 2 Translational Tissue Engineering Center, Johns Hopkins University School of Medicine, Baltimore, MD, United States; 3 Department of Chemical and Biomolecular Engineering, Johns Hopkins University, Baltimore, MD, United States; 4 Department of Immunology, University of Pittsburgh School of Medicine, Pittsburgh, PA, United States; 5 Center for Systems Immunology, University of Pittsburgh School of Medicine, Pittsburgh, PA, United States; 6 Graduate Program in Microbiology and Immunology, University of Pittsburgh School of Medicine, Pittsburgh, PA, United States; 7 Department of Biomedical Engineering, Johns Hopkins University School of Medicine, Baltimore, MD, United States; 8 Department of Computational and Systems Biology, University of Pittsburgh School of Medicine, Pittsburgh, PA, United States; 9 Bloomberg Kimmel Institute for Cancer Immunotherapy, Johns Hopkins University, Baltimore, MD, United States; 10 Sidney Kimmel Comprehensive Cancer Center, Johns Hopkins University, Baltimore, MD, United States; 11 Department of Oncology, Johns Hopkins University School of Medicine, Baltimore, MD, United States; 12 Department of Ophthalmology, Johns Hopkins University School of Medicine, Baltimore, MD, United States; 13 Department of Molecular Microbiology & Immunology, Johns Hopkins University Bloomberg School of Public Health, Baltimore, MD, United States

**Keywords:** adoptive cell transfer, antibody, autoimmune disease, cell engineering, immunocytokine, interleukin-2, protein engineering, regulatory T cells

## Abstract

**Introduction:**

Interleukin-2 (IL-2) is a multifunctional cytokine that potently expands regulatory T cells (Tregs) and thus has potential in mitigating autoimmune diseases and promoting transplant tolerance. However, the cytokine’s concurrent activation of effector lymphocytes coupled with its short serum half-life limit therapeutic use. Previous efforts have overcome these challenges by fusing IL-2 to an engineered anti-IL-2 antibody denoted F5111, which selectively directs IL-2 towards Tregs over effector lymphocytes. The resulting molecule, denoted the F5111 immunocytokine (IC), potently and specifically expands Tregs, but its bulky size and bivalency limit diffusion and tissue penetration, while its dual-chain format complicates gene delivery and stable expression from cells.

**Methods:**

Here, we engineered a miniaturized version of F5111 IC (termed miniF5111 IC), comprising IL-2 fused to a single chain variable fragment (scFv) of the F5111 antibody. We optimized the topology of miniF5111 IC and performed biophysical, signaling, and functional studies to interrogate its immune activity.

**Results:**

Binding studies revealed that miniF5111 IC mimics the receptor binding bias of the full-length F5111 IC and, consistent with these results, cell signaling studies showed that miniF5111 IC preferentially stimulates Tregs over effector lymphocytes. *In vivo*, miniF5111 IC mediated selective Treg expansion with a reduced serum half-life compared to the full-length F5111 IC. Finally, we expressed miniF5111 IC from engineered Tregs and showed that stable expression of this molecule led to prolonged cell persistence and sustained FOXP3 expression following adoptive transfer.

**Discussion:**

Taken together, our findings position miniF5111 IC as a versatile platform to selectively target and activate specific immune cell subsets, demonstrating its potential as a next-generation therapeutic to treat autoimmune disorders and prevent transplant rejection.

## Introduction

Regulatory T cells (Tregs) play a critical role in immune tolerance and have been recognized as a compelling therapeutic candidate for autoimmune diseases and transplant tolerance. Although the adoptive transfer of ex vivo-expanded Tregs has shown promise in preclinical and early-phase clinical studies, complex manufacturing, stability, and safety challenges have hindered broad clinical translation of this approach (1–3). Limitations with the adoptive transfer workflow have stimulated interest in endogenous or transplanted antigen-specific Treg-selective *in vivo* expansion strategies that can potentially be integrated to enrich the immunotherapeutic landscape for autoimmune disease.

Interleukin-2 (IL-2) is a pleiotropic cytokine that supports the expansion and sustains the function of both immune effector cells (i.e., effector T cells and natural killer [NK] cells) and Tregs through its binding to two functionally distinct receptor complexes with different cytokine affinities. High-affinity binding (equilibrium dissociation constant [K_D_]≈10 pM) is mediated by a trimeric receptor complex composed of IL-2Rα (CD25), IL-2Rβ (CD122), and the common gamma chain (γ_c_[CD132]), whereas intermediate-affinity binding (K_D_≈1 nM) is mediated by a dimeric complex containing only IL-2Rβ and γ_c_ (4–6). Although IL-2Rβ and γ_c_mediate IL-2-mediated signaling, IL-2Rα (designated as the low-affinity IL-2 receptor) is not a signaling component but rather augments cellular sensitivity to IL-2 (6). Engagement of IL-2 with either of its signaling receptor complexes triggers the Janus kinase-signal transducer and activator of transcription (JAK-STAT) pathway and subsequent changes in gene expression that modulate immune responses (7, 8). Tregs constitutively express high levels of IL-2Rα, rendering them ≈100-fold more responsive to IL-2 than resting immune effector cells, which express low levels of IL-2Rα. This differential sensitivity forms the basis for the use of low-dose IL-2 therapy for selective expansion of Tregs for therapeutic applications in autoimmune disease and transplantation medicine (9–11). Despite its inherent proclivity towards Tregs, IL-2 also stimulates immune effector cells, and its short serum half-life can be a pharmacokinetic limitation (9, 12, 13).

Multiple protein engineering strategies have been employed to improve the selectivity and *in vivo* persistence of the IL-2 cytokine. One strategy, first pioneered by Boyman et al., relies on IL-2/anti-IL-2 antibody complexes to bias cytokine activity toward specific immune subsets (14). Building on this work, Trotta et al. identified the human antibody F5111.2, which binds human IL-2 and sterically blocks cytokine binding to IL-2Rβ while also allosterically reducing cytokine affinity towards IL-2Rα (15). Activation of immune cells by the IL-2/F5111.2 complex is gated by the dissociation of cytokine from the antibody complex upon its binding to the IL-2Rα subunit, which enables signaling through the IL-2Rβ and γ_c_chains. Consequently, the IL-2/F5111.2 complex preferentially stimulates IL-2Rα^High^ cells (i.e., Tregs) over immune effector cells. This mechanism parallels the exchange/release model also described for other Treg-biased anti-IL-2 antibodies such as JES6–1 and UFKA-20 (16, 17). Although Treg-biased IL-2/anti-IL-2 antibody complexes have shown robust preclinical promise in ameliorating autoimmune conditions and promoting transplant tolerance (17–23), clinical administration of cytokine/antibody complexes requires careful optimization of dosing ratios and complex dissociation leads to disease-promoting immune effector cell activation by the released IL-2 (13, 16, 24).

**Figure 1:**
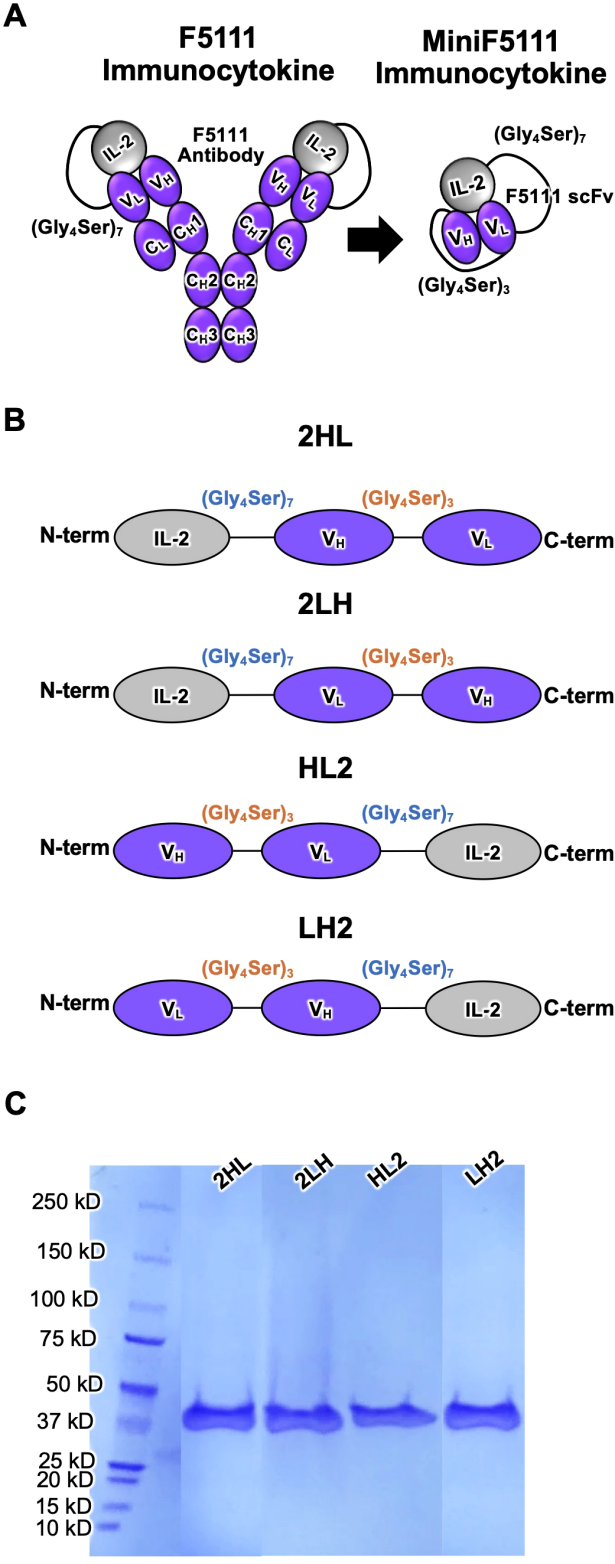
Design and purification of miniF5111 immunocytokines. **(A)** Schematic representation of full-length F5111 immunocytokine (IC) and the corresponding miniF5111 IC design, in which IL-2 is fused to the F5111 antibody single-chain variable fragment (scFv) via a flexible (Gly_4_Ser)_7_ linker **(B)** Diagram of 4 miniF5111 IC orientations (2HL, 2LH, HL2, and LH2), depicting the relative positions of IL-2 and scFv domains. **(C)** SDS-PAGE analysis of purified miniF5111 IC variants confirming expected molecular weights and purity.

To address these limitations, Vandyke et al. developed a single-agent fusion protein, F5111 immunocytokine (IC) (25), which covalently links IL-2 to F5111, the parent antibody of the affinity-matured F5111.2 antibody developed by Trotta et al. (15). The intramolecularly assembled IC selectively expands Tregs and confers protection in mouse models of colitis and immune checkpoint inhibitor-induced diabetes mellitus (15). However, the large size and bivalency of F5111 IC may limit its diffusion and tissue penetration, which can be a barrier to effective disease targeting in tissue-specific autoimmune disorders (26). In addition, the dual-chain format of F5111 IC complicates gene delivery or stable expression from engineered cells. Here, we describe the engineering and characterization of a miniaturized F5111 IC (miniF5111 IC), in which IL-2 is genetically fused to a single-chain variable fragment (scFv) of the F5111 antibody (**Figure 1**). In previous work, the scFv format has demonstrated several pharmacological advantages compared to full-length IgGs, including superior diffusion arising from the smaller size, monovalent binding, and improved biodistribution (27–31). In addition, the miniF5111 IC format is amenable to fusion with other biologics, such as antibody drugs, which could enable tissue-specific targeting and expanded functionalities. Furthermore, use of the single-chain miniF5111 IC as opposed to the dual-chain F5111 IC format would simplify gene delivery and stable expression from engineered cells by reducing both the length and complexity of the encapsulated genetic material. Notably, previous studies have fused IL-2 to scFv constructs to selectively target the cytokine towards tumor-associated antigens (28, 32); however, the innovation of this study lies in the development of an intramolecularly assembled cytokine/scFv fusion protein. We designed and characterized multiple versions of miniF5111 IC with different fusion orientations to identify an optimized construct. Cellular and animal studies revealed that our lead construct selectively expands Tregs and supports the stable *in vivo* persistence of engineered Tregs. Collectively, these data establish miniaturized ICs as a potential class of next-generation immunotherapies for the treatment of autoimmune diseases.

## Materials and methods

### Cell lines and media

Human embryonic kidney (HEK) Expi293™ cells (Thermo Fisher Scientific) were maintained in Expi293™ Expression Medium (Gibco) containing 2 U/mL penicillin-streptomycin (Gibco), shaking at 37 °C in a humidified incubator with 5% CO_2_.

YT-1 human NK cells were cultured in RPMI-1640 medium (Gibco) supplemented with 10% heat-inactivated fetal bovine serum (FBS, Gibco), 1% GlutaMAX (Gibco),100 U/mL penicillin-streptomycin (Gibco), 1% Minimum Essential Medium Non-Essential Amino Acids solution (MEM NEAA, Gibco), 1% sodium pyruvate, and 1.5% 4-(2-hydroxyethyl)-1 piperazineethanesulfonic acid (HEPES, Gibco). 5KC cells were a kind gift from M. Nakayama and cultured in IMDM media (Gibco) supplemented with 10% FBS (Gemini Bio) and 100 U/mL penicillin-streptomycin (Gibco). Platinum-E (Cell Biolabs) cells (33) were cultured in DMEM media (Corning) supplemented with 10% FBS (Gemini Bio), 50 µM beta-mercaptoethanol, 10 mM HEPES (Corning), 1% MEM NEAA solution (Corning), 1% GlutaMAX (Gibco), 25 µg/mL Gentamycin (Gibco), 10 µg/mL Blasticidin (Gibco), 1 µg/mL Puromycin (Thermo Scientific), and 100 U/mL penicillin-streptomycin (Gibco). Cells were maintained at 37 °C in a humidified incubator containing 5% CO_2_ and passaged every 2–3 days to maintain growth.

For signaling studies, deidentified healthy human blood donor samples were obtained from Johns Hopkins School of Medicine. Human peripheral blood mononuclear cells (PBMCs) were isolated using Ficoll-Paque Plus (GE Healthcare) gradient and SepMate™ tubes (Stemcell Technologies), per manufacturer recommendations, followed by incubation with ACK lysis buffer (Gibco) for removal of red blood cells. The isolated PBMCs were then resuspended in RPMI-1640 (Gibco) supplemented with 10% heat-inactivated FBS (Gibco), 1% GlutaMAX (Gibco), and 100 U/mL penicillin-streptomycin (Gibco). Cells were kept chilled on ice until use in same-day signaling assays.

For *in vivo* Treg transfer studies, primary murine Tregs were cultured in ImmunoCult XF T cell expansion medium (StemCell Technologies) supplemented with 50 nM rapamycin (Thermo Fisher), 1000 U/mL human IL-2 (R&D Systems), and 100 U/mL penicillin-streptomycin (Gibco).

### Protein purification and expression

The F5111 antibody and F5111 IC were recombinantly expressed in human embryonic kidney (HEK) Expi293™ cells (Thermo Fisher Scientific), as previously described (25). HEK Expi293™ cells were cultured to a density of 3.0×10^6^ cells/mL and diluted to 1.5×10^6^ cells/mL prior to transfection. Transient transfection of F5111 was performed using gWiz mammalian expression plasmids encoding the human immunoglobulin G (IgG) 1 lambda F5111 antibody heavy chain (HC) and light chain (LC) constructs. Transient transfection of F5111 IC was conducted using plasmids encoding the F5111 HC and the F5111 LC fused to IL-2 (residues 1-133) at the N-terminus, connected by a flexible (Gly_4_Ser)_7_ linker (25). DNA (1 mg/L culture) was diluted to 0.01 mg/mL in Opti-MEM medium (Gibco), quickly vortexed, and then combined with polyethyleneimine Max (PEI MAX, Polysciences, 1 mg/L culture) which was diluted to 0.0533 mg/mL. The DNA and PEI MAX was then briefly vortexed and incubated for 10 minutes at room temperature. The DNA/PEI MAX mixture (100 mL/L culture) was then added to the HEK Expi293™ cells, and the cells were incubated at 37°C with shaking for 5 days. One day after transfection, valproic acid, sodium propionate, and glucose (Sigma) were added to final concentrations of 5 mM, 6.9 mM, and 46 mM, respectively. Secreted proteins were purified from culture supernatants via Protein G affinity chromatography (Thermo Fisher Scientific), followed by size-exclusion chromatography (SEC) on an ÄKTA™ fast protein liquid chromatography (FPLC) system equipped with a Superdex 200 column (Cytiva), equilibrated with HEPES-buffered saline (HBS; 150 mM NaCl, 10 mM HEPES, pH 7.3). Protein purity (>99%) was assessed by sodium dodecyl sulfate polyacrylamide gel electrophoresis (SDS-PAGE) analysis.

The miniF5111 IC plasmid DNA constructs (also in the gWiz mammalian expression vector) were generated by fusing human IL-2 (residues 1–133) at either the N- or C-terminus of the single-chain variable fragment (scFv) of the F5111 antibody, connected by a flexible 35-amino acid (Gly_4_Ser)_7_ linker. The variable heavy and light chains within the scFv were connected by a 15-amino acid (Gly_4_Ser)_3_ linker. The miniControl IC was constructed analogously to miniF5111 IC substituting the published variable heavy and light chain sequences of the FITC-E2 antibody (34). Sequences for all full-length and mini IC constructs are presented in **Supplementary Table S1**. Constructs were cloned into the gWiz expression vector (Genlantis) and expressed via transient transfection of HEK Expi293™ cells, as detailed for F5111 antibody and F5111 IC. Secreted miniF5111 IC proteins were purified from conditioned supernatants using Nickel nitrilotriacetic acid (Ni-NTA) affinity chromatography followed by size-exclusion chromatography (SEC) on an ÄKTA™ fast protein liquid chromatography (FPLC) system using a Superdex 200 column equilibrated in HBS. Purity was confirmed by SDS-PAGE analysis.

Human IL-2 (amino acids 1–133), IL-2Rα ectodomain (amino acids 1–217), and IL-2Rβ ectodomain (amino acids 1–214), each containing a C-terminal hexahistidine tag, were cloned into the gWiz mammalian expression vector and were produced and purified from HEK Expi293™ cells, as described for the mini ICs. For preparation of biotinylated IL-2, IL-2Rα, and IL-2Rβ, a C-terminal biotin acceptor peptide (BAP) GLNDIFEAQKIEWHE sequence was included in the plasmid DNA construct. Following expression and Ni-NTA chromatography purification of BAP sequence-containing proteins, the extracted cytokine and receptors were biotinylated with the soluble BirA ligase enzyme in 0.05 M Bicine pH 8.3, 10 mM ATP, 10 mM magnesium acetate, and 50 μM biotin (Avidity) for 1 hour at room temperature, followed by an overnight incubation at 4 °C. Excess biotin was then removed by SEC on an ÄKTA^™^ FPLC (Cytiva) instrument using a Superdex 200 column (Cytiva). Protein biotinylation and purity were verified with a streptavidin gel-shift assay via SDS-PAGE analysis.

### YT-1 cell signaling studies

Approximately 2×10^5^ IL-2Rα^+^ YT-1 (35) or IL-2Rα^+^ YT-1 (36) human NK cells were plated in each well of a 96-well plate and resuspended in 20 μL of RPMI complete medium containing serial dilutions of either IL-2 or IC. Cells were stimulated for 15 minutes at 37 °C and immediately fixed by addition of paraformaldehyde (Electron Microscopy Sciences) to a final concentration of 1.6% and incubated for 10 minutes at room temperature. Permeabilization of cells was achieved by resuspension in 200 μL of ice-cold 100% methanol (MilliporeSigma) for 30 minutes on ice. Fixed and permeabilized cells were washed twice with PBSA and incubated with anti-pSTAT5 AlexaFluor 647 (pY694, BD Biosciences 562076, 1:50) diluted in 20 μL of PBSA for 1 hour at room temperature. Cells were then washed twice in PBSA and analyzed on a CytoFLEX flow cytometer (Beckman Coulter). Dose-response curves were fitted to a logistic model and maximum values (E_Max_) and half maximal effective concentration (EC_50_) values were calculated using GraphPad Prism data analysis software version 10.0.3 (GraphPad) after subtraction of the mean fluorescence intensity (MFI) of unstimulated cells and normalization to the maximum signal intensity. Experiments were conducted in duplicate and performed at least twice with similar results.

### Human PBMC isolation and signaling studies

Approximately 2×10^6^ PBMCs were plated in each well of a 96-well plate immediatelyfollowing isolation. Viability (>99%) was confirmed by staining with LIVE/DEAD Fixable Aqua deadcell stain (Invitrogen) diluted 1:1000 in PBS for 15 minutes at 4 °C. Cells were resuspended in RPMI complete medium containing serial dilutions of the appropriate treatment. Cells were incubated with the prescribed treatments for 20 min at 37°C, and immediately fixed and permeabilized with Transcription Factor Phospho (TFP) Buffer kit (BD Pharmingen), per manufacturer recommendations. Cells were then stained for 1 hr at room temperature with the following antibodies in 1×TFP Perm/Wash buffer (BD Pharmingen): APC-eFluor780 anti-human CD3 (clone UCHT1, Invitrogen, 1:50); PerCP-Cy5.5 anti-human CD4 (clone SK3, BD Pharmingen, 1:100); BV605 anti-human CD8 (clone SK1, Biolegend, 1:50); BV421 anti-human IL-2Rα (clone M-A251, BD Horizon, 1:100); Alexa Fluor 488 anti-human CD127 (clone eBioRDR5, Invitrogen, 1:50); PE anti-human FOXP3 (clone 236A/E7, BD Pharmingen, 1:50); and Alexa Fluor 647 anti-STAT5 (clone pY694, BD Biosciences, 1:50). Cells were then washed twice and resuspended in PBSA buffer. Data were collected on an Attune NxT flow cytometer (Invitrogen) and analyzed using FlowJo software version 10.10.0 (FlowJo, LLC). Tregs were gated as CD3^+^CD4^+^IL-2Rα^High^FOXP3^High^ cells, CD8^+^ T cells were gated as CD3^+^CD4^-^CD8^+^ cells, and conventional CD4^+^ T cells (Tconvs) were gated as CD3^+^CD4^+^FOXP3^-^ cells (**Supplementary Figure S2**). pSTAT5 dose-response curves were fitted to a logistic model and E_Max_ and EC_50_ values were calculated using GraphPad Prism data analysis software version 10.0.3 (GraphPad) after subtraction of the MFI of unstimulated cells. PBMC activation experiments were conducted twice with similar results using independent donors.

### Biolayer interferometry binding measurements

Biotinylated human IL-2, IL-2Rα and IL-2Rβ were immobilized to streptavidin-coated tips for analysis on an Octet Red96 biolayer interferometry instrument (Sartorius). Less than 5 signal units (nm) of IL-2 or each protein was immobilized to minimize mass transfer effects. PBSA (phosphate-buffered saline [PBS] pH 7.2 containing 0.1% BSA) was used for all dilutions and as the dissociation buffer. Tips were exposed to serial dilutions of IL-2, F5111 antibody, or ICs in a 96-well plate for 300 s. Dissociation was then measured for 450 s. Surface regeneration for all interactions was conducted using 30 s exposure to 0.1 M glycine pH 2.7. Normalized equilibrium binding curves were obtained by plotting the response value at the end of the association phase for each sample dilution and normalizing to the maximum value. Equilibrium binding curves were fitted and equilibrium dissociation constant (K_D_) values were determined using GraphPad Prism data analysis software v10.0.0 (GraphPad), assuming all binding interactions are first order. Kinetic analyses were performed using GraphPad Prism data analysis software v10.0.0, assuming a 1:1 binding model. Experiments were conducted at least twice with similar results.

### *In vivo* immune cell subset expansion

For relative immune cell subset expansion studies, female 8-week-old female C57BL/6J mice were obtained from the Jackson Laboratory and housed at Johns Hopkins University animal facilities. Mice were injected intraperitoneally with a dose representing the molar equivalent of 0.075 (low dose) or 0.375 mg/kg (high dose) IL-2 of mini ICs, F5111 IC, or Control IC in 250 μL of PBS, on days 1, 2, 3, and 4. Mice were randomly distributed into experimental groups, with approximately equal average body weight across groups. On day 5 (24 hr after the last injection), mice were sacrificed by inhalation of isofluorane followed by cervical dislocation, and spleens were harvested for cell isolation. Single-cell suspensions were prepared by homogenization (GentleMACS Dissociator, Miltenyi Biotec) and subjected to ACK red blood cell lysis buffer (Gibco).

Isolated splenocytes were resuspended in modified PBE buffer (PBS pH 7.4 with 2.5% fetal calfserum [FCS], 20 mmol ethylenediaminetetraacetic acid [EDTA]). The cells from each spleen were thensplit into 3 groups, which were each stained in modified PBE buffer for 30 min on ice with each ofthe following panels including live/dead stain FVD-eF780 (ThermoFisher Scientific, 1:700): Tregpanel, which included eFluor 450 anti-mouse CD3 (clone 17A2, ThermoFisher Scientific, 1:40), Super Bright 702 anti-mouse CD4 (clone RM4.5, ThermoFisher Scientific, 1:100), PerCP anti-mouse CD45 (clone 30-F11, BD Biosciences, 1:300), and APC anti-mouse IL-2Rα (IL-2Rα, clone PC61.5, ThermoFisher Scientific, 1:300); CD8^+^ T cell panel, which included PE-Cy7 anti-mouse CD3 (clone 145-2C11, ThermoFisher Scientific, 1:40), Super Bright 702 anti-mouse CD4 (clone RM4.5, ThermoFisher Scientific, 1:100), PerCP anti-mouse CD45 (clone 30-F11, BD Biosciences, 1:300), BD Horizon V500 anti-mouse CD8 (clone 53-7.62, BD Biosciences, 1:80); and NK cell panel, which included BD Horizon V500 anti-mouse CD3 (clone 500A2, BD Biosciences, 1:30), PerCP anti-mouse CD45 (clone 30-F11, BD Biosciences, 1:300), FITC anti-mouse CD49b (clone DX5, ThermoFisher Scientific, 1:50), and APC anti-mouse CD161 (clone PK136, ThermoFisher Scientific, 1:125). Cells were then washed twice and fixed in Fixation/Permeabilization Buffer (BD Biosciences) for 1 hr on ice. The Treg panel was stained with PE anti-mouse/rat FOXP3 (clone FJK-16s, ThermoFisher Scientific, 1:100) for 30 min on ice, followed by two final washes and resuspension in modified PBE buffer. Samples were analyzed on a 5-laser (355/407/488/561/633 nm) Cytek Aurora spectral flow cytometer (Cytek). Unmixing was performed using SpectroFlo software (Cytek). The percentages of splenic lymphocytes that were Tregs (CD3^+^CD4^+^IL-2Rα^+^FOXP3^+^) and Tconvs (CD3^+^CD4^+^FOXP3^-^) were determined from the Treg panel; the percentages of splenic lymphocytes that were CD8^+^ T cells (CD3^+^CD4^-^CD8^+^) were determined from the CD8^+^ T cell panel; the percentages of splenic lymphocytes that were NK cells (CD3^-^CD49b^+^CD161^+^) were determined from the NK cell panel (**Supplementary Figure S4**). Data were analyzed using FlowJo software version 10.10.0 (FlowJo, LLC). The total number of splenocytes was determined by analyzing each single cell suspension prepared from spleen on an automated cell counter (Cellaca MX, Nexcelom Bioscience, USA). Average ratios of the relative expansion of the indicated subsets were plotted using GraphPad Prism data analysis software version 10.0.3 (GraphPad). Experiments were performed in triplicate and conducted twice with similar results.

### Pharmacokinetic studies

Human IL-2 and ICs were labeled using 10-fold molar excess of N-hydroxysuccinimide (NHS)-Rhodamine (ThermoFisher Scientific), following the manufacturer’s protocol. Non-reacted NHS-rhodamine was removed using a Zeba Spin Desalting Column (ThermoFisher Scientific), equilibrated in HBS. Protein concentration and degree of labeling were calculated via spectrophotometry, according to the manufacturer’s protocol.

Prior to treatment, ~20 μL of blood was collected via tail excision of female 8-week-old C57BL/6J mice (The Jackson Laboratory). Mice were housed at Johns Hopkins University animal facilities. Retro-orbital injections of IL-2 or ICs (~0.36 mg/kg IL-2 equivalence, ~7 mg IL-2 total/mouse) diluted in 100 μL of PBS were administered to mice. Blood was collected from each mouse tail at 15 minutes, 30 minutes,1 hour, 2 hours, 4 hours, 6 hours, 12 hours, 24 hours, 48 hours, 72 hours, 96 hours, and 120 hours. At each time point, blood was diluted (1:10) in PBS/EDTA (150 mM NaCl, 12 mM Na2HPO4, 5 mM EDTA, pH 8) and centrifuged at 10,000×g for 5 minutes. The plasma was collected, added to a 96 well black clear-bottom plate and stored at 4 °C for later analysis. After all samples were collected, fluorescence (Excitation/Emission: 540/590 nm) was measured on a Varioskan LUX Multimode Microplate Reader instrument (ThermoFisher Scientific). Blood samples collected before treatment were used for background subtraction. Standard curves were generated from fluorescent measurements of rhodamine-labeled proteins at concentrations ranging from 1 μM to 17 pM (3-fold dilutions), and this standard curve was used to determine protein concentration of the collected samples. Serum half-life was calculated using a 2-phase decay model in GraphPad Prism data analysis software version 10.0.3 (GraphPad).

### Retroviral engineering of miniF5111 IC into Tregs and *in vitro* and *in vivo* analysis

MiniF5111 IC was cloned into the pMSCV-ZsGreen-2A retroviral vector between the NotI and BamHIrestriction sites (**Supplementary Figure S6**). pMSCV vectors were packaged using Platinum-E (Cell Biolabs) cells (33). Briefly, Platinum-E cells were transfected with the pMSCV-ZsGreen-2A-miniF5111 shuttle plasmids using TransIT-293 (Mirus) following manufacturer’s instructions. Viral supernatants were collected 48 hours post-transfection, filtered through a 0.45 μm filter (Millipore Sigma), and concentrated using RetroX Concentrator (Takara) according to the manufacturer’s protocol. Concentrated virus was aliquoted and stored at -80 °C until use in transductions.

Female 6–10 week-old non-obese diabetic (NOD) mice (NOD/ShiLtJ; strain 001976) werepurchased from Jackson Laboratory and housed at the University of Pittsburgh animal facilities. Micewere euthanized by CO_2_ asphyxiation followed by cervical dislocation, after which spleens were isolated and mechanically dissociated to generate single cell suspensions. CD4^+^ T cells were enriched by magnetic negative selection (StemCell Technologies) and stained with Zombie yellow (Biolegend, 1:500) PerCP/Cy5.5 anti-mouse CD4 (clone GK1.5, BioLegend, 1:200), and PE anti-mouse IL-2Rα antibody (clone PC61, BioLegend, 1:200) for subsequent fluorescence-activated cell sorting on a FACSAria Special Order Research Product (BD Biosciences). Sorted Tregs (Zombie yellow^-^CD4^+^IL-2Rα^High^) were stimulated with anti-CD3/CD28 antibody-coated beads (Dynabeads, Thermo Fisher) at a bead-to-cell ratio of 3:1 and cultured for 3 days. Tregs were transduced with concentrated retrovirus 3 days after isolation and stimulation using RetroNectin (Takara) coated plates according to the manufacturer’s protocol. Tregs were plated on virus-bound wells in the presence of protamine sulfate (5 mg/mL), rapamycin (50 nM), and IL-2 (1000 U/mL). After 3 days of transduction, anti-CD3/CD28 antibody-coated beads were magnetically removed, and cells were either used for injections or cultured in ImmunoCult XF T cell expansion medium supplemented with 100 U/mL penicillin-streptomycin under reduced IL-2 conditions (300 U/mL) and without rapamycin. Secretion of miniF5111 IC was measured from cell supernatant (obtained by centrifuging cells for 3 minutes at 600×g) using His-Tag ELISA kits (Abcam) 4 days post-transduction according to manufacturer’s protocols (**Supplementary Figure S6**).

Immediately prior to adoptive transfer on Day 1 (**Supplementary Figure S7**), cells were analyzed on the Attune NxT flow cytometer (Thermo Fisher Scientific) toquantify ZsGreen expression. To quantify the FOXP3 expression of cells on the day of adoptivetransfer (Day 1), an aliquot of cells was stained with Zombie yellow (Biolegend, 1:500) for 20 minutes at room temperature, protected from light. Cells were washed with FACS buffer (PBS lacking Ca^2+^ and Mg^2+^ with 2% FBS), then fixed and permeabilized using the True-Nuclear Transcription Factor Buffer Set (Biolegend), according to the manufacturer’s protocol. For permeabilization staining, cells were incubated with PE-Cy7 anti-mouse FOXP3 antibody (clone FJK-16s, Thermo Fisher, 1:100) diluted in permeabilization buffer for 30 minutes at room temperature, protected from light. Day 1 FOXP3 staining studies were analyzed on an Aurora flow cytometer (Cytek) (**Supplementary Figure S7**).

Female 8-10-week-old NOD.SCID (NOD.Cg-Prkdcscid/J; strain 001303) mice were purchased from theJackson Laboratory and maintained at the University of Pittsburgh. Mice received 1x10^6^mock or miniF5111 IC-engineered Tregs via retro-orbital injections using an insulin syringe (BD). Blood samples were taken via retro-orbital bleeds 7, 14, 23, 30, and 35 days post-injection. Blood volumes were collected into 1.5 mL microcentrifuge tubes coated with 0.5M EDTA. Normalized volumes of blood were centrifuged at 1500 × g for 15 minutes at 4 °C. For the detection of ZsGreen and surface protein expression, blood pellets were resuspended with RBC-lysis buffer (BioLegend) for 15 minutes at room temperature. Zombie yellow (Biolegend, 1:500)) staining was then performed for 20 minutes at room temperature, protected from light. Cells were washed with FACS buffer (PBS lacking Ca^2+^ and Mg^2+^ with 2% FBS) and resuspended in surface antibody cocktail, consisting of APC anti-mouse CD4 (clone GK1.5, BioLegend, 1:200), pacific blue anti-mouse CD3 (clone 17A2, BioLegend, 1:200), PE anti-mouse IL-2Rα (clone PC61, BioLegend, 1:200). Surface stains were incubated for 20 minutes on ice, protected from light. Cells were washed with FACS buffer and resuspended in FACS buffer prior to acquisition on an Attune NxT flow cytometer (Thermo Fisher Scientific) (**Supplementary Figure S7**). ZsGreen detection was not possible using the following intracellular staining protocol.Therefore, these additional steps were used to determine the FOXP3 expression of the cells inparallel. Following viability and surface staining, cells were fixed and permeabilized using the True-Nuclear Transcription Factor Buffer Set according to the manufacturer’s protocol (BioLegend). For permeabilization staining, cells were incubated with PE-Cy7 anti-mouse FOXP3 (clone FJK-16s, Thermo Fisher, 1:100) antibody diluted in permeabilization buffer for 30 minutes at room temperature, protected from light. FOXP3 staining studies were acquired on an Aurora flow cytometer (Cytek) (**Supplementary Figure S8**). Flow cytometry data were analyzed using FlowJo software version 10.10.0 (FlowJo, LLC). Data were plotted using GraphPad Prism data analysis software version 10.0.3 (GraphPad).

## Results

### Iterations of miniF5111 IC showed differential expression yields and quality

F5111 IC was engineered into a cytokine-scFv fusion protein format by designing 4 iterations of miniF5111 IC that encompassed all possible molecular topologies, with the goal of identifying the configuration that best enhances Treg bias (**Figure 1**). Human IL-2 was fused to either the N- or C-terminus of the scFv of the F5111 antibody (15) using a 35-amino acid (Gly_4_Ser)_7_ linker, as was found to be necessary to allow intramolecular assembly of the cytokine and antibody within F5111 IC (25). The scFv comprised the variable heavy and light chains of the F5111 antibody, separated by a standard 15-amino acid (Gly_4_Ser)_3_ linker (**Figure 1****;** **Supplementary Table 1**). Each construct was named based on its orientation (2HL, 2LH, HL2, and LH2). A control construct (denoted miniControl IC) was generated by substituting the variable heavy and light chain antibody domains within miniF5111 IC (in 2LH format) with those of an irrelevant isotype-matched anti-fluorescein isothiocyanate (FITC) antibody (clone FITC-E2) (34).

MiniF5111 IC variants were produced via transient transfection of human embryonic kidney (HEK) Expi293™ cells and purified from cell supernatants via Ni-NTA chromatography followed by size-exclusion chromatography (SEC). All 4 miniF5111 IC constructs and miniContol IC were successfully expressed and exhibited the expected molecular weights, as confirmed by sodium dodecyl sulfate polyacrylamide gel electrophoresis (SDS-PAGE) analysis (**Figure 1**; **Supplementary Figure S1**). Size-exclusion chromatography traces revealed varying levels of oligomerization, with amonodisperse peak at the expected elution volume for each construct (**Supplementary Figure S1**). Amongst the miniF5111 IC variants, miniF5111 IC HL2 exhibited a significantly higherproportion of oligomer relative to monomer; thus, this construct was excluded from further study. MiniF5111 IC 2LH also showed elevated oligomerization; however, optimization of the purification protocol substantially reduced oligomer formation, yielding a predominantly monomeric species (**Supplementary Figure S1**). Yields for each of the miniF5111 IC constructs ranged from 1-3.5 mg/L (**Supplementary Figure S1**).

### MiniF5111 IC 2LH demonstrates selectivity towards IL-2Rα^High^ immune cells

**Figure 2:**
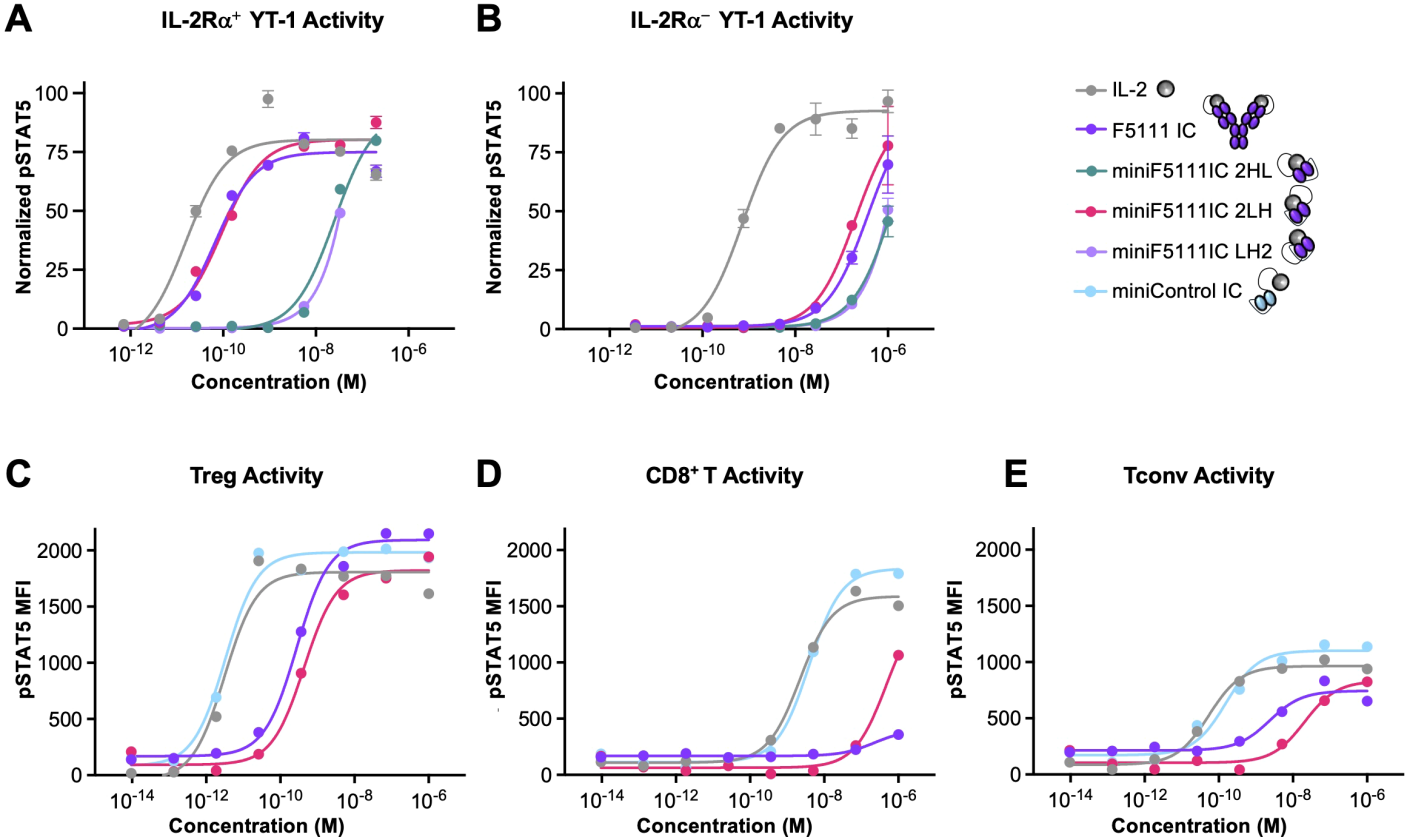
2LH orientation of miniF5111 IC exhibits IL-2Rα-dependent selectivity in human immune cells. **(A, B)** STAT5 phosphorylation response in **(A)** IL-2Rα^+^ and **(B)** IL-2Rα^+^ YT-1 cell lines following stimulation with IL-2, F5111 IC, miniF5111 IC variants, or miniControl IC. Data represent mean ± SD (n=2). **(C–E)** STAT5 phosphorylation responses in stimulated human peripheral blood mononuclear cell (PBMC) subsets, including **(C)** Tregs, **(D)** CD8^+^ T cells, and **(E)** Tconvs. Data show mean and is representative of at least two independent experiments.

IL-2 dependent STAT5 phosphorylation (pSTAT5) by each of the miniF5111 IC variants was assessed in YT-1 human NK cells (35) with or without IL-2Rα expression (36). The YT-1 cell system serves as a model for IL-2 signaling on IL-2Rα^High^ Tregs versus IL-2Rα^Low^ immune effector cells. On IL-2Rα^+^ YT-1 cells, miniF5111 IC 2LH elicited robust activation comparable to that induced by the full-length F5111 IC, which was ≈5-fold less potent than signaling induced by untethered IL-2 (**Figure 2****;** **Supplementary Table S2**). By contrast, miniF5111 IC variants 2HL and LH2 showed significantly attenuated potency (≈1500-fold and ≈15,000-fold reduced potency compared to free IL-2, respectively) on IL-2Rα^+^ YT-1 cells. On IL-2Rα^+^ cells, both miniF5111 IC 2LH and F5111 IC showed significantly reduced potency (>250-fold) relative to untethered IL-2 (**Figure 2**), demonstrating bias towards IL-2Rα^+^ cell signaling compared to the native IL-2 cytokine. miniF5111 IC variants 2HL and LH2 showed ≈2000-fold weaker and ≈7000-fold weaker potency relative to untethered IL-2, respectively, on IL-2Rα^+^ YT-1 cells. Collectively, these findings indicate that amongst the miniF5111 IC variants, only the 2LH construct effectively activates cells with the desired bias, conferring a selectivity towards IL-2Rα^+^ cell activation equivalent to that of F5111 IC. Incidentally, miniF5111 IC fuses IL-2 to the F5111 scFv in an analogous topology to that of F5111 IC, with the cytokine linked to the N-terminus of the variable light chain of the antibody. Due to its functional mimicry of F5111 IC, miniF5111 IC 2LH was selected for further analysis.

To confirm Treg-selective activation by miniF5111 IC within a mixed human immune cell population, pSTAT5 signaling induced by this fusion protein on human peripheral blood mononuclear cells (PBMCs) was evaluated. On Tregs, miniF5111 IC 2LH was nearly as potent as the full-length F5111 IC, and both ICs were ≈90-fold less potent than untethered IL-2 and miniControl IC, as expected due to the requirement for antibody dissociation to enable signaling (**Figure 2**; **Supplementary Table S2**) (25). As previously reported, F5111 IC did not induce activation of CD8^+^ T cells (25), and miniF5111 IC 2LH similarly did not activate CD8^+^ T cells below a concentration of 1μM (**Figure 2**). In contrast, untethered IL-2 and miniControl IC potently activated CD8^+^ T cells with half maximal effective concentration (EC_50_) values in the single-digit nM range (**Supplementary Table S2**). On CD4^+^ effector T cells (Tconvs), F5111 IC and miniF5111 IC 2LH led to significantly less potent activation and attenuated maximal response (E_Max_) values compared with untethered IL-2 and miniControl IC (**Figure 2**). Interestingly, miniF5111 IC exhibited ≈10-fold weaker potency than F5111 IC on Tconv cells (**Supplementary Table S2**). Taken together, these PBMC studies confirm that miniF5111 IC 2LH preserves the selectivity of F5111 IC, preferentially activating Tregs over effector cell subsets. Based on these results, we proceeded with miniF5111 IC 2LH in subsequent studies, and we hereafter denote this molecule miniF5111 IC.

### Intramolecular assembly of miniF5111 IC allows binding to IL-2Rα while blocking IL-2/IL-2Rβ interactions

**Figure 3:**
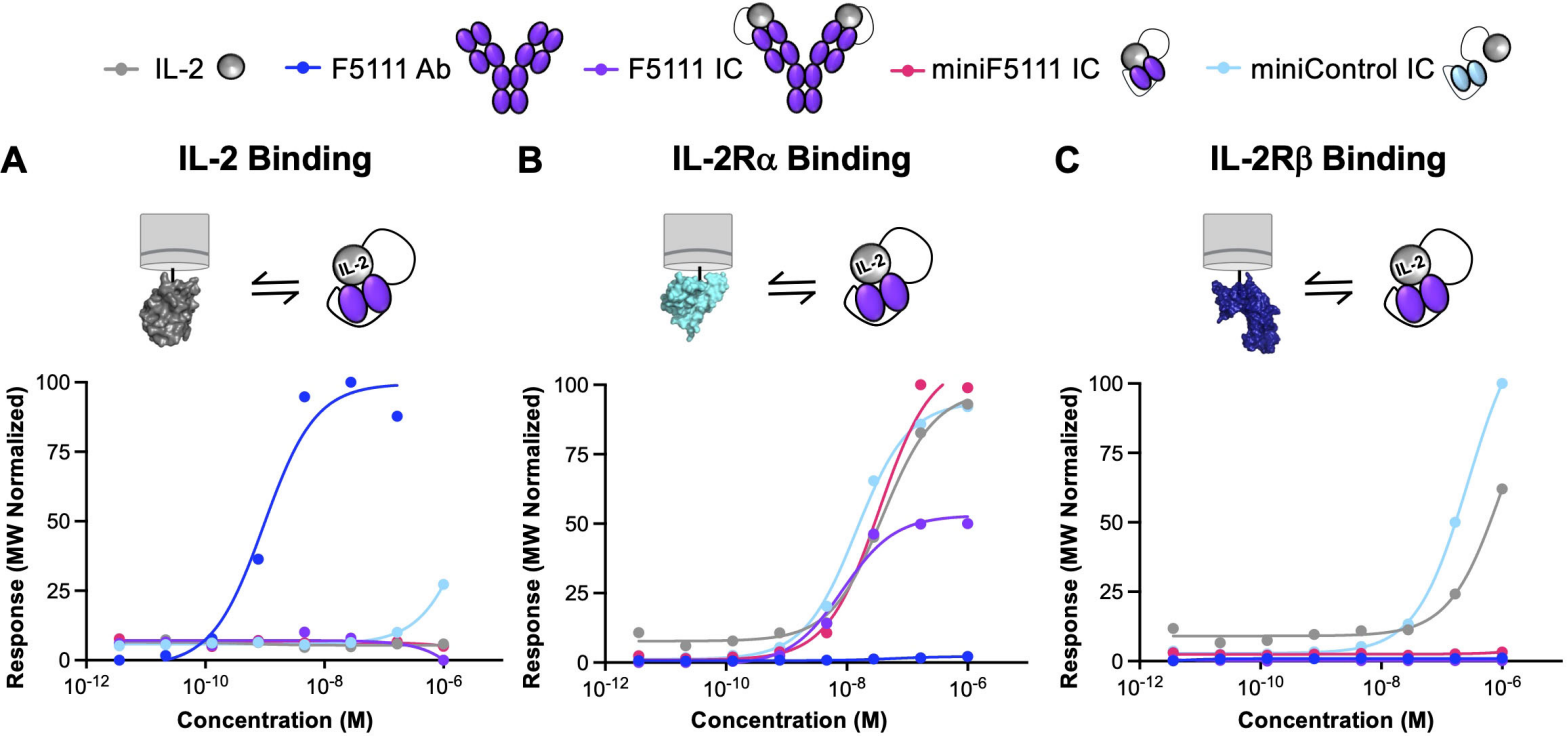
MiniF5111 IC demonstrates intramolecular assembly and IL-2Rα-biased binding. **(A–C)** Equilibrium binding of IL-2, F5111 antibody (Ab), F5111 IC, miniF5111 IC, and miniControl IC to immobilized **(A)** IL-2, **(B)** IL-2Rα, and **(C)** IL-2Rβ, as measured by biolayer interferometry.

To evaluate the intramolecular assembly and functional activity of miniF5111 IC, the binding affinities against immobilized human IL-2, IL-2Rα, and IL-2Rβ were measured using biolayer interferometry. Binding of an F5111-containing IC to IL-2 would be abrogated if the cytokine is intramolecularly engaged with the antibody. Consistent with this hypothesis, the F5111 antibody bound IL-2, whereas the full-length F5111 IC and miniF5111 IC did not, indicating proper intramolecular assembly of the cytokine and antibody chains within these ICs (**Figure 3**; **Supplementary Figure S3****;** **Supplementary Table S3**). Neither untethered IL-2 nor miniControl IC showed binding to IL-2, as expected. Because the IL-2Rα binding site on IL-2 remains accessible when the cytokine is bound to F5111, we expected miniF5111 IC to retain binding to IL-2Rα. Indeed, miniF5111 IC displayed comparable IL-2Rα affinity to untethered IL-2 and miniControl IC (**Figure 3**; **Supplementary Figure S3**). The IL-2Rα binding affinity of miniF5111 IC was ≈4-fold weaker than that of the full-length F5111 IC, mainly due to an accelerated dissociation rate (k_off_) (**Supplementary Table S3**). This reduced affinity is likely due to avidity effects resulting from bivalent presentation of IL-2 in the full-length F5111 IC. As F5111 blocks the IL-2Rβ binding epitope on IL-2, we anticipated that properly assembled F5111-containing ICs would not interact with the IL-2Rβ subunit. As expected, miniF5111 IC and F5111 IC did not engage IL-2Rβ, whereas untethered IL-2 and miniControl IC bound to IL-2Rβ with similar affinities (**Figure 3**; **Supplementary Figure S3****;** **Supplementary Table S3**). Overall, these binding analyses confirm the proper intramolecular assembly of IL-2 and F5111 antibody domains within miniF5111 IC and elucidate the rationale for its bias towards IL-2Rα^High^ Tregs.

### MiniF5111 IC selectively expands Tregs *in vivo*

**Figure 4:**
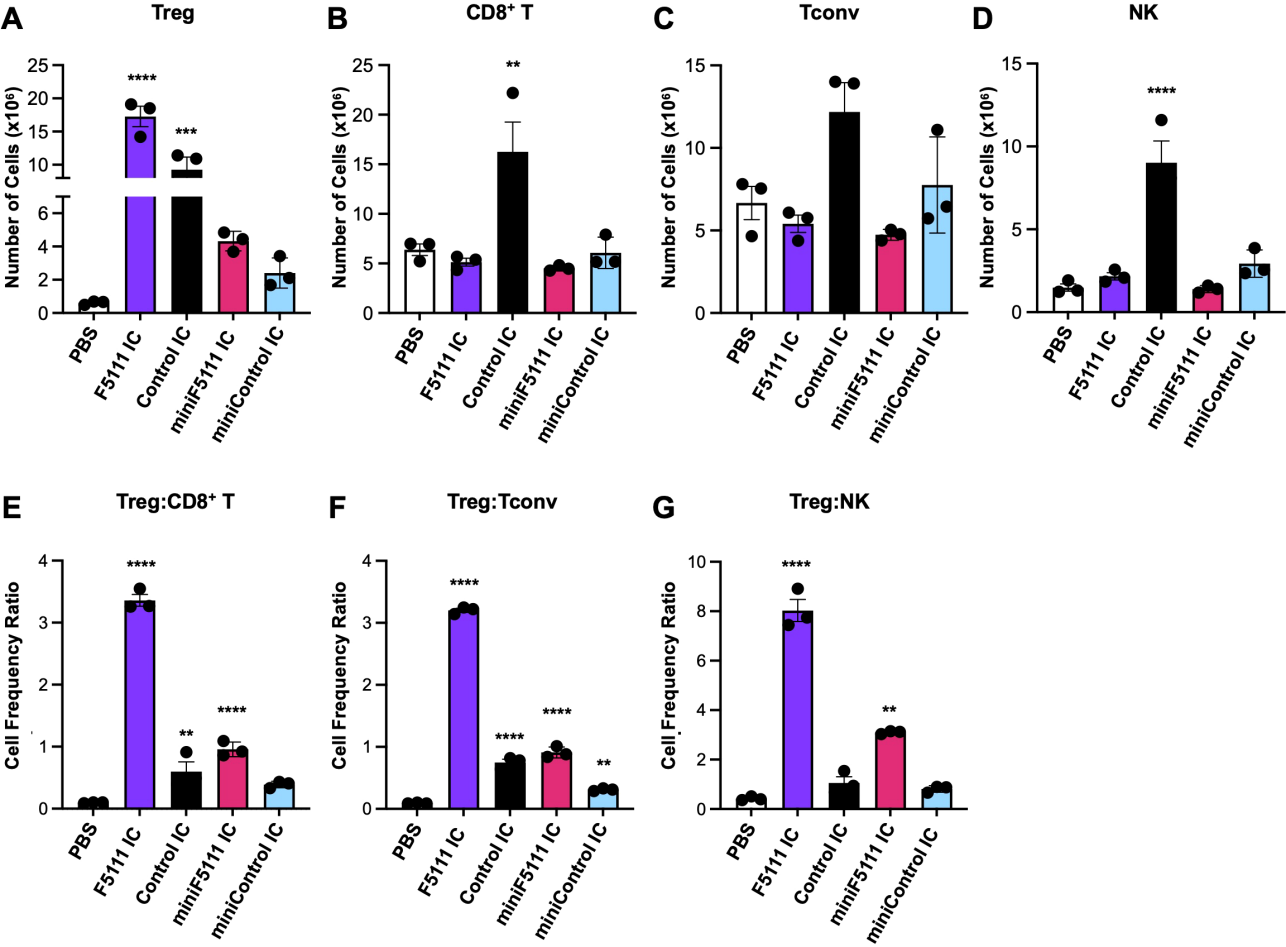
MiniF5111 IC selectively expands regulatory T cells *in vivo*. **(A–G)** C57BL/6 mice (n=3 per group) were treated daily for 4 consecutive days with PBS, F5111 IC (1.5 μg IL-2 equivalent), Control IC (1.5 μg IL-2 equivalent), miniF5111 IC (7.5 μg IL-2 equivalent), and miniControl IC (7.5 μg IL-2 equivalent). Spleens were harvested 24 hours after the last injection for flow cytometry analysis. **(A–D)** Total number of **(A)** Treg, **(B)** CD8^+^T, **(C)** Tconv, and **(D)** NK cells. **(E–G)** Ratios of Treg cells to **(E)** CD8^+^ T, **(F)** Tconv, and **(G)** NK cells. Data represent mean ± SD (n=3); Statistical significance was determined by one-way ANOVA with Tukey’s multiple comparison test and is noted only for comparisons to PBS. *p<0.05, **p<0.01, ***p<0.001, ****p<0.0001. All statistical comparisons are presented in **Supplementary Table S5**.

To confirm that the *in vitro* bias of miniF5111 IC towards activation ofIL-2Rα^High^ cells translates into biased activation of Tregs *invivo*, C57BL/6J mice were treated with various full-length and mini ICs, and harvested splenocytes were analyzed by flow cytometry to quantify Tregs, effector T cells, and NK cells (**Supplementary Figure S4**). We initially administered miniF5111 IC at equivalent doses compared to the full-lengthF5111 IC; however, no statistically significant changes were observed in the absolute counts of theTreg, CD8^+^ T, Tconv, or NK cell populations compared to treatment with either PBS or miniControl IC (**Supplementary Figure S5**, **Supplementary Table S5**). We hypothesized that this lack of immune activity was due to the shorter half-life of miniF5111 IC versus the full-length F5111 IC, since the absence of an Fc domain precludes neonatal Fc receptor (FcRn)-mediated recycling (37), allowing for rapid renal clearance for this 47 kD molecule (30, 38). As such, we repeated the study including an additional cohort with higher dosing of miniF5111 IC and miniControl IC (5× that of the full-length F5111 IC and Control IC). Under these higher dosing conditions, miniF5111 IC resulted in a 7-fold increase in Tregs compared to the saline control cohort and a 2-fold increase in Tregs compared to both the miniControl IC and low-dose miniF5111 IC treatment cohorts (**Figure 4**). Although this increase was still modest compared to that induced by F5111 IC or even Control IC (and did not reach statistical significance), this result was expected given the lack of an Fc domain, and this feature allows for the possibility of targeting and restricting availability of miniF5111 IC to locally expand Tregs rather than imposing systemic and persistent immune effects. Importantly, miniF5111 IC, like F5111 IC, did not expand immune effector subsets and, in fact, led to decreases in CD8^+^ T cells (0.3-fold reduction), Tconvs (0.3-fold reduction), and NK cells (0.1-fold reduction) relative to saline-treated control mice (**Figure 4B**). Consistent with these results, the Treg: CD8^+^ T, Treg: Tconv, and Treg: NK cell ratios following miniF5111 IC treatment relative to those of saline-treated control mice demonstrated the Treg-selective activity of the miniaturized IC (**Figure 4E**). In contrast, miniControl IC had no effect on the Treg: CD8^+^ T or Treg: NK cell ratios and a more limited effect compared to miniF5111 IC on the Treg: Tconv ratio, confirming the Treg bias induced by the F5111 scFv within miniF5111 IC. As with cell counts, the effect of miniF5111 IC on Treg to effector T and NK cell ratios was less pronounced than that of the full-length F5111 IC, presumably due to the reduced serum persistence of the miniaturized IC (**Figure 4E**). Altogether, these studies demonstrate that miniF5111 IC drives selective expansion of Tregs *in vivo* while avoiding activation of effector T and NK cells.

### MiniF5111 IC exhibits intermediate serum half-life relative to IL-2 and F5111 IC

**Figure 5:**
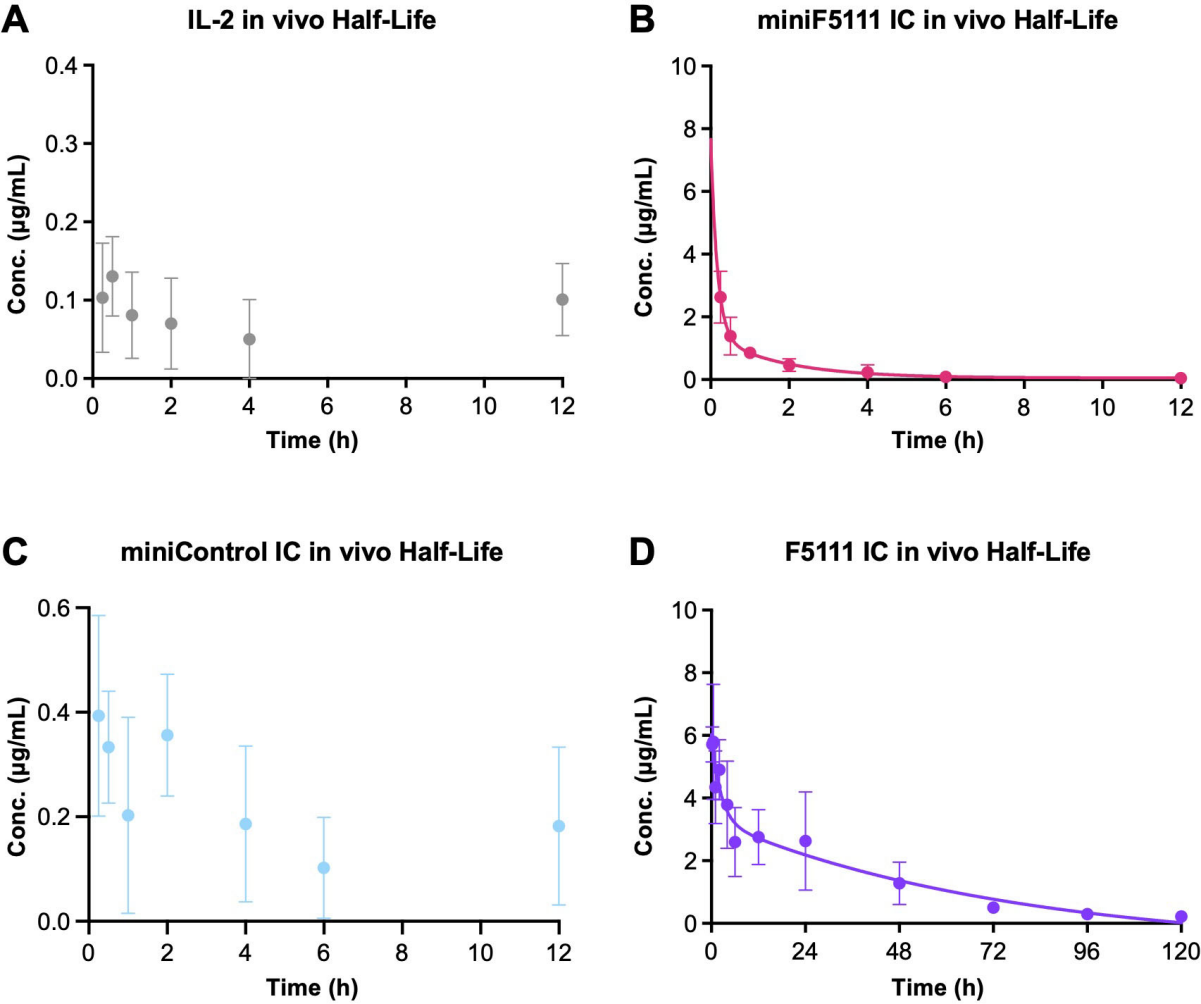
MiniF5111 IC exhibits intermediate serum half-life relative to IL-2 and full-length F5111 IC. **(A–D)** Pharmacokinetic analysis of serum clearance profiles in C57BL/6 mice following retro-orbital administration of **(A)** IL-2, **(B)** miniF5111 IC, **(C)** miniControl IC, and **(D)** F5111 IC, all at an equivalent IL-2 dose of ~0.4 mg/kg. Data show mean ± SD (n=5) and are representative of two independent experiments.

Based on the results of *in vivo* immune cell subset expansion studies, we conjectured that miniF5111 IC was significantly less persistent *in vivo* compared to the full-length F5111 IC owing to the lack of an Fc domain (37). We therefore conducted pharmacokinetic analyses in C57BL/6J mice to compare the serum clearance profile of miniF5111 IC to those of untethered IL-2, miniControl IC, and F5111 IC (**Figure 5**). Whereas untethered IL-2 cleared quickly due to its known rapid renal clearance (39), and the cytokine was nearly undetectable within 15 minutes of injection (**Figure 5**), serum concentrations of miniF5111 IC exhibited a two-phase decay with an initial fast half-life of ~7.5 minutes, followed by a slow half-life of ~1.6 hours (**Figure 5**). Interestingly, miniControl IC behaved similarly to untethered IL-2, with extremely rapid clearance and barely measurable serum concentrations within 15 minutes of injection (**Figure 5**). As anticipated, the Fc domain-bearing full-length F5111 IC cleared much more gradually than miniF5111 IC, with a fast half-life of ~1.6 hours and a slow half-life of ~50 hours (**Figure 5**). Collectively, these pharmacokinetic studies confirm that miniF5111 IC possesses an intermediate half-life compared to untethered IL-2 and the full-length F5111 IC, a property that alters its *in vivo* behavior compared to these other molecules, and which could potentially be leveraged to modulate spatiotemporal availability of the cytokine.

### Transduction of miniF5111 IC into Tregs enhances *in vivo* persistence while maintaining FOXP3 stability

We envision that the truncated *in vivo* half-life of miniF5111 IC compared tofull-length F5111 IC combined with its compact single-chain format can be exploited to allow forlocalized, transient delivery from cells. In particular, we aimed to program Tregs to secrete miniF5111 IC to sustain their own growth and also deliver this molecule in the context of adoptive cell transfer. To this end, we first sought to establish that miniF5111 IC can be expressed from Treg cells. We transduced Tregs from non-obese diabetic (NOD) with a retroviral vector encoding both miniF5111 IC and a ZsGreen reporter, separated by a self-cleaving P2A peptide (**Supplementary Figure S6**). 4 days post-transduction, we detected miniF5111 IC secretion from Tregs transduced withthis vector (termed miniF5111 IC Tregs), but not mock-transduced Tregs (termed Mock Tregs) (**Supplementary Figure S6**).

**Figure 6:**
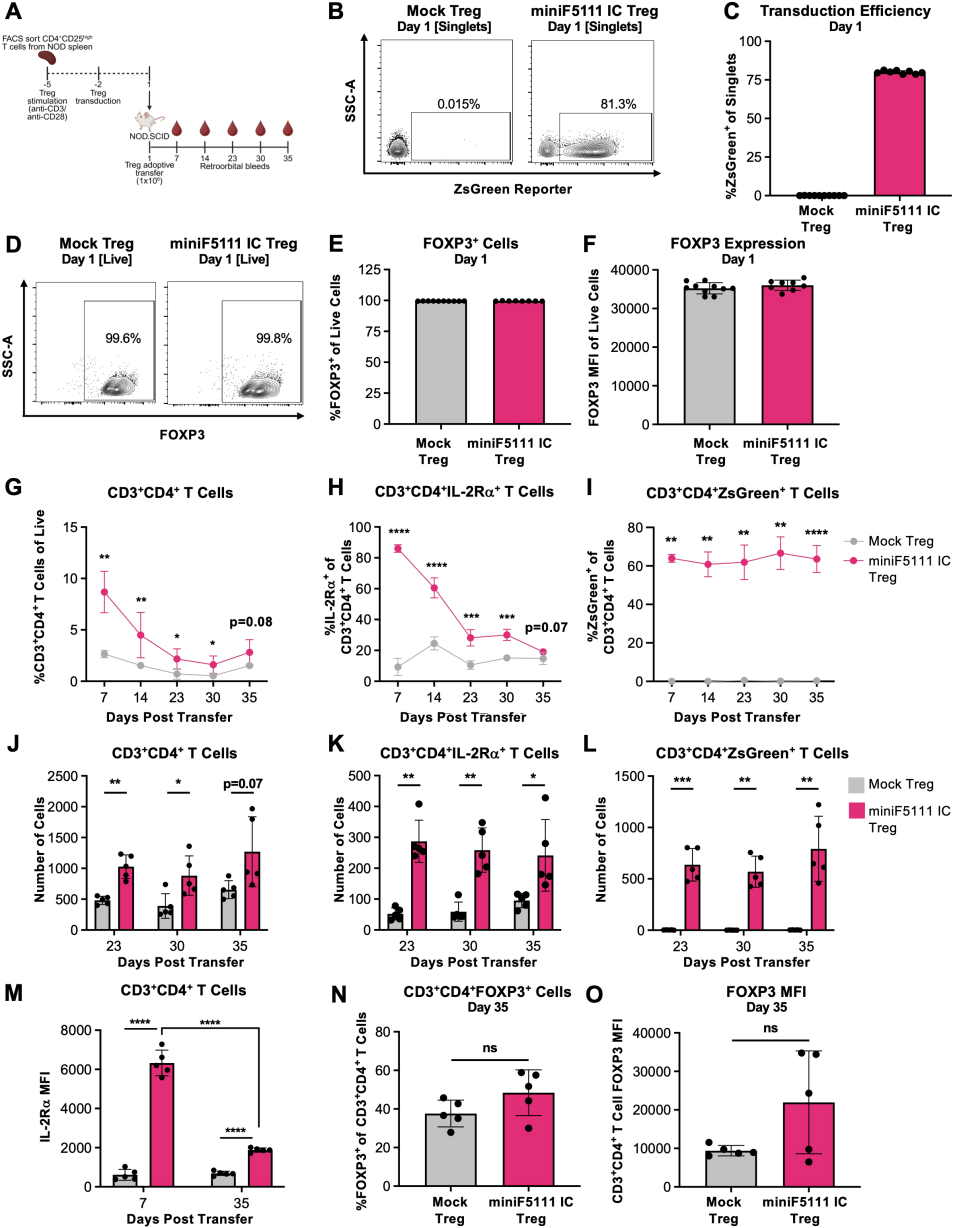
Transduction of miniF5111 IC into Tregs enhances *in vivo* persistence while maintaining FOXP3 stability following adoptive transfer. **(A)** Schematic depicting the experimental workflow for Treg engineering and *in vivo* persistence study. IL-2Rα^High^ CD4^+^ Tregs were FACS sorted from NOD splenocytes, stimulated with anti-CD3/anti-CD28 antibody beads in the presence of IL-2 and rapamycin, and then retrovirally transduced to express the miniF5111 constructs. Following an adoptive transfer of engineered Tregs, NOD-SCID mice were bled weekly for analysis of Tregs by flow cytometry. **(B)** Representative contour plots of ZsGreen reporter, indicating transduction levels of engineered Tregs immediately prior to adoptive transfer (Day 1). **(C)** Flow cytometry-based quantification of ZsGreen reporter frequency, indicating transduction levels of engineered Tregs immediately prior to adoptive transfer (Day 1). **(D)** Representative contour plots of FOXP3 expression of engineered Tregs immediately prior to adoptive transfer (Day 1). **(E, F)** Flow cytometry-based quantification of frequency **(E)** and magnitude **(F)** of FOXP3 expression engineered Tregs immediately prior to adoptive transfer (Day 1). **(G-O)** Analysis of transferred Tregs from RO bleeds by flow cytometry days 7–35 post-adoptive transfer, depicting **(G)** frequency and **(J)** total number of live CD3^+^CD4^+^ T cells from retro-orbital bleeds 7–35 days post-adoptive transfer, **(H)** frequency and **(K)** total number of live CD3^+^CD4^+^IL-2Rα^+^ T cells from retro-orbital bleeds 7–35 days post-adoptive transfer, **(I)** frequency and **(L)** total number of live CD3^+^CD4^+^ZsGreen^+^ T cells from retro-orbital bleeds performed 7–35 days post-adoptive transfer, **(M)** IL-2Rα MFI of live CD3^+^CD4^+^ T cells from retro-orbital bleeds performed 7–35 days post-adoptive transfer, **(N)** frequency and **(O)** magnitude of FOXP3 expression of live CD3^+^CD4^+^ T cells 35 days post-adoptive transfer. Data in panels **(C)**, **(E)**, and **(F)** represent mean ± SD of technical replicates (n=10 for Mock Treg cohort and n=8 for miniF5111 IC cohort) from one biological replicate. Data in panels **(G–O)** represent mean ± SD (n=5). Data normality was evaluated with the Shapiro-Wilk test and statistical significance was determined with Mann-Whitney or Welch’s *t*-test as appropriate, according to normality. *p<0.05, **p<0.01, ***p<0.001, ****p<0.0001. All statistical comparisons are presented in **Supplementary Table S5**.

Having demonstrated the capacity to secrete miniF5111 IC from Tregs, we adoptively transferred these transduced Tregs into NOD.SCID mice to evaluate cell persistence and phenotype (**Figure 6**. Prior to transfer, miniF5111 IC Tregs exhibited robust ZsGreen reporter expression (**Figure 6B**; **Supplementary Figure S7**), confirming successful transduction. Moreover, miniF5111 IC Tregs maintained high levels of the critical Treg transcription factor FOXP3 relative to Mock Tregs (**Figure 6D**; **Supplementary Figure S7**), demonstrating preservation of the Treg phenotype at baseline. Following adoptive transfer, mice that received miniF5111 IC Tregs displayed significantly increased frequencies of transferred CD4^+^ T cells (defined as CD3^+^CD4^+^ cells) as well as IL-2Rα-expressing CD4^+^ T cells over time compared with mice that received mock Tregs (**Figure 6G**; **Supplementary Figure S8**). Of note, significantly higher persistence of mice with transferred miniF5111 IC Tregs versus mock Tregs was observed for 30 days post transfer, demonstrating the durability of miniF5111 IC expression. Transferred cells also maintained ZsGreen reporter expression, as expected (**Figure 6**). Enhanced persistence of total and IL-2Rα-expressing transferred CD4^+^ T cells was also reflected in overall CD4^+^ and CD4^+^ IL-2Rα^+^ T cell counts on Days 23, 30 and 35 (**Figure 6J**). Also, the number of ZsGreen reporter-expressing cells remained stable for 35 days after miniF5111 IC Treg transfer (**Figure 6**), further corroborating the sustained survival of these cells post-transfer. Interestingly, expression levels of IL-2Rα on miniF5111 IC-transduced adoptively transferred T cells decreased over the 35-day study, but still remained significantly higher than those on mock transduced adoptively transferred T cells (**Figure 6**). To determine whether FOXP3 expression was stable in Tregs post-transfer, mice were sacrificed on Day 35 for intracellular FOXP3 quantification. We observed comparable frequency and magnitude of FOXP3 expression within the CD4^+^ T cell population between mice that received miniF5111 IC Tregs and those that received mock Tregs (**Figure 6N**; **Supplementary Figure S8**), illustrating the retention of Treg phenotype for these cells over the course of the study. Collectively, these results demonstrate that miniF5111 IC can be expressed from Tregs and that adoptively transferred Tregs engineered to produce this protein show superior *in vivo* persistence while maintaining IL-2Rα and FOXP3 expression.

## Discussion

In this study, we have designed a miniaturized single‐chain immunocytokine (miniF5111 IC) to address two key translational challenges of full-length ICs. The parent F5111 IC biases IL-2 signaling to Tregs and is a promising therapeutic for autoimmune disease (25), but its bivalent, full-length format has critical limitations including poor tissue diffusion, suboptimal tissue penetration, and challenges in gene delivery and cell‐based expression (26, 27, 40). In contrast, miniF5111 IC is significantly smaller (~47 vs ~180 kD), monovalent, and can be readily expressed from engineered cells, properties that will enable broad applications of this molecule in gene and cell therapies.

Our biophysical and functional data support intramolecular assembly and Treg-biased activity of miniF5111 IC in the 2LH orientation (**Figure 1**) which recapitulates the topology of F5111 IC, where IL-2 is fused at the N-terminus of thevariable light chain (25). Strikingly, other orientations of miniF5111 IC (2HL, HL2 and LH2) were not optimal, with HL2 showing a high degree of oligomerization (**Supplementary Figure S1**) and 2HL and LH2 having suboptimal activity on IL-2Rα^+^ YT-1 cells (**Figure 2**), likely due to linker length restrictions and/or linker interference that could also drive oligomerization (25). In particular, the 35-amino acid linker between IL-2 and F5111 was optimized for the 2LH format (25). Thus, it is possible that this linker length could be further optimized to improve intramolecular assembly and function of other miniF5111 IC geometries. This configuration reproduces the binding properties of the full-length IC (25), which does not bind to IL-2Rβ but retains IL-2Rα binding (**Figure 3B**), and this highly selective receptor engagement is key for Treg specificity. Furthermore, these binding results are consistent with previous structural studies that showed that F5111 antibody sterically blocks IL-2/IL-2Rβ, but not IL-2/IL-2Rα engagement (16). Based on these binding studies and corresponding cell signaling activity, we postulate that the 2LH format best recapitulates the receptor‐exchange mechanism enacted by F5111 IC, in which IL-2 is transiently released from the antibody to bind the high‐affinity trimeric receptor, which has previously been described for Treg-biasing anti-IL-2 antibodies including JES6-1, UFKA-20, and F5111 (15, 17, 19). We note that the expression and manufacturability of miniF5111 IC in 2LH format can be further optimized by tuning linker length and/or composition.

Remarkably, despite miniF5111 IC being monovalent, it retained the same Treg‐biased signaling phenotype as the full-length F5111 IC in both YT-1 and PBMC signaling studies (**Figure 2**). This unexpected finding would indicate that the two arms of the F5111 IC work independently. However, we also found that miniF5111 IC had a ~4-fold lower IL-2Rα equilibrium binding affinity than F5111 IC, likely due to loss of avidity effects that contribute to bivalent IL-2 presentation (**Figure 3****;** **Supplementary Table S3**). Therefore, we conclude overall that avidity losses affect receptor binding, but do not have an impact at the level of signaling. This observation can be explained by the fact that the reduced IL-2Rα affinity for miniF5111 IC is primarily due to reduction in k_off_; therefore we hypothesize that the similar k_on_ rates for miniF5111 IC and full-length F5111 IC lead to similar signaling outcomes for these molecules.

*In vivo*, miniF5111 IC induced Treg expansion and significantly increased Treg:effector T cell ratios relative to the saline-treated control mice (**Figure 4**). The relatively lower Treg: effector T cell ratios induced by miniF5111 IC compared to the full-length F5111 IC is likely reflective of its shorter serum half-life (**Figure 5**) and can be overcome with higher dosing (**Supplementary Figure S5**). The intermediate pharmacokinetic profile of miniF5111 IC relative to IL-2 and F5111 IC could have therapeutic advantages. As demonstrated by previous work, the shorter *in vivo* half-life of miniF5111 IC compared to the full-length IC could allow for localized Treg expansion while limiting systemic exposure, which could potentially interfere with healthy immune clearance (27, 30, 41). It will be important to fully characterize the biodistribution and pharmacodynamic properties of miniF5111 IC in future studies. Moreover, the half-life of miniF5111 IC can be tuned, for example, by fusion to serum albumin or Fc domains (42–46). Importantly, even at high, IL-2-equivalent doses, miniF5111 IC induces minimal activation of effector T cells, highlighting the large safety window of this molecule and suggesting that the dosing tolerance of this molecule will be amenable for biomedical uses.

We showed that the single-chain design of miniF5111 IC allowed for its stable incorporation into engineered Tregs, enabling these cells to constitutively secrete the molecule into their immediate microenvironment. This secretion creates a supportive cytokine environment that further enhances IL-2Rα-dependent signaling on neighboring and self-producing Tregs. In adoptive Treg cell transfer studies, miniF5111 IC‐expressing Tregs demonstrated significantly improved persistence and sustained FOXP3 expression relative to mock Tregs, which rapidly diminished in the absence of exogenous IL-2 (**Figure 6**). Interestingly, the percentage of ZsGreen reporter-positive cells remained elevated throughout the experiment (**Figure 6**) despite the observed monotonic decrease in Treg numbers (**Figure 6G**). We also noted a decrease in IL-2Rα expression on miniF5111 IC-transduced adoptively transferred cells over time (**Figure 6**), and the mechanism for this reduced expression will be important to explore in future work. While we were unable to directly quantify the *in vivo* production of miniF5111 IC by the adoptively transferred Tregs, presumably due to rapid clearance of the construct from circulation (**Figure 5**), the significantly prolonged survival of miniF5111 IC-transduced Tregs over mock-transduced Tregs between days 7 and 30 after adoptive transfer demonstrates the sustained *in vivo* expression of the construct during this time period. Future preclinical studies will be required to assess *in vivo* expression kinetics and determine whether expression of the construct is associated with durable suppressive function of the engineered Tregs. We note that the early survival advantage we observed in ZsGreen^+^ miniF5111 IC-transduced Tregs followed by a subsequent decrease in cell numbers is in fact strikingly similar to the kinetics previously described for adoptively transferred Tregs constitutively expressing a mutated version of IL-2 (47). Overall, our *in vivo* adoptive Treg transfer studies suggest that miniF5111 IC can create a self-perpetuating, localized signaling environment that enhances Treg survival and lineage fidelity, and which can be leveraged to improve the persistence and therapeutic efficacy of Treg cell therapies.

A broad range of engineering approaches have been undertaken to directly bias human IL-2 activity through mutation of the cytokine, resulting in generation of muteins. In the context of Treg bias, mutein strategies have focused on significantly weakening IL-2 binding to IL-2Rβ and/or γ_c_ with partial or no disruption to IL-2Rα binding, so that these molecules increase their dependence on IL-2Rα expression and therefore signal primarily on IL-2Rα^High^ Tregs (48–51). Contrasting with mutein approaches, miniF5111 IC encodes the wild-type human IL-2 sequence, reducing the potential for creating neo-epitopes that can potentially drive immunogenicity. Furthermore, miniF5111 IC achieves its Treg selectivity through a gated molecular exchange mechanism, in which the F5111 antibody prevents IL-2 activity until IL-2Rα^High^ Tregs induce dissociation of the antibody to allow signaling to occur with native kinetics (15). In contrast, IL-2 muteins are permanently reduced in their intrinsic signaling capacity due to their altered receptor binding properties, particularly those that drastically impair binding to the IL-2Rβ and/or γ_c_ chains. Thus, miniF5111 IC allows more flexibility in controlling the system by introducing a gated signaling model that can be turned on or off rapidly.

Furthermore, miniF5111 IC achieves its Treg selectivity through a gated molecular exchange mechanism, in which the F5111 antibody prevents IL-2 activity until IL-2Rα^High^ Tregs induce dissociation of the antibody to allow signaling to occur with native kinetics (15). In contrast, IL-2 muteins are permanently reduced in their intrinsic signaling capacity due to their altered receptor binding properties, particularly those that drastically impair binding to the IL-2Rβ and/or γ_c_ chains. Thus, miniF5111 IC allows more flexibility in controlling the system by introducing a gated signaling model that can be turned on or off rapidly.

Functionally, *in vitro* signaling studies of the IL-2-REH mutein on human peripheral blood mononuclear cells (PBMCs) showed ≈1000-fold increased potency on human Tregs relative to CD8^+^ T cells (49). Similar results were observed in our PBMC studies, wherein miniF5111 IC also showed ≈1000-fold increased potency on Tregs relative to CD8^+^T cells (**Figure 2C**). Interestingly, we did not detect a difference in signaling potency between the full-length F5111 IC and miniF5111 IC; thus, the *in vitro* bias of the parental F5111 IC was also comparable to that of IL-2-REH. For *in vivo* studies, the IL-2-REH mutein induced ≈3-fold expansion of Tregs relative to saline control (49), whereas miniF5111 induced ≈7-fold expansion of Tregs relative to saline control (**Figure 4**), suggesting that intramolecularly gated IL-2 delivery achieves at least equivalent levels of Treg bias relative to IL-2 mutein designs. With respect to pharmacokinetics, IL-2 muteins rapidly clear from circulation, similar to the IL-2 cytokine (**Figure 5**), thus, therapeutic approaches with these molecules have employed Fc fused versions that take advantage of FcRn-mediated recycling to improve serum persistence (48, 50, 51). miniF5111 IC has a slightly longer half-life (~1.5 hours) when compared to untethered IL-2, which clears within minutes (**Figure 5A**), resulting in serum persistence that is intermediate between untethered muteins and Fc-fused IL-2 muteins. This increased serum persistence may account for the slightly higher Treg expansion orchestrated by miniF5111 IC compared to untethered IL-2-REH.

The modular single-chain design of miniF5111 IC is broadly applicable to translational use cases such as gene‐encoded delivery (52–54), incorporation into chimeric antigen receptor (CAR) Treg platforms (55–57), or fusion to multispecific antibodies for tissue-targeted Treg recruitment. Collectively, these properties position miniF5111 IC as a highly versatile new scaffold for targeted immune regulation with broad therapeutic utility in the context of autoimmune diseases, transplantation, and chronic inflammatory conditions.

## Data Availability

The datasets presented in this study can be found in online repositories. The names of the repository/repositories and accession number(s) can be found below: https://www.ncbi.nlm.nih.gov/genbank/, Accession numbers: PX644988-PX644997.
