# Supplementary material for: Miniaturized IL-2/anti-IL-2 immunocytokines selectively activate and support the in vivo persistence of regulatory T cells
Source: Front Immunol. 2026 Mar 9;17:1755812. doi: 10.3389/fimmu.2026.1755812 (PMC13007364; doi:10.3389/fimmu.2026.1755812)
Supplement: Supplementary file 1 [file DataSheet1.pdf]

Figure S1.

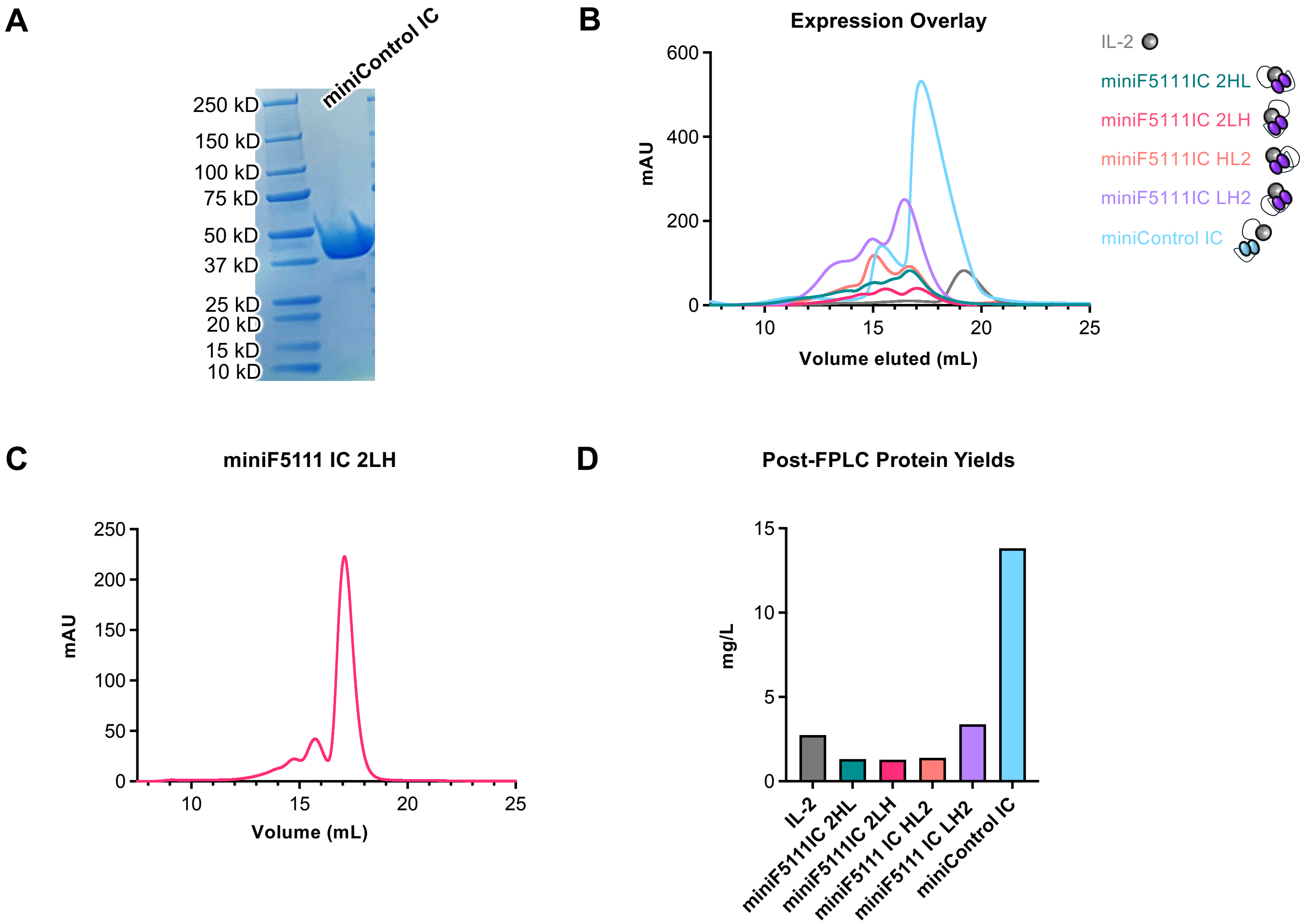

A
miniControl IC
250 kD
150 kD
100 kD
75 kD
50 kD
37 kD
25 kD
20 kD
15 kD
10 kD
B
Expression Overlay
mAU
600
400
200
0
10
15
20
25
Volume eluted (mL)
IL-2
miniF5111IC 2HL
miniF5111IC 2LH
miniF5111IC HL2
miniF5111IC LH2
miniControl IC
C
miniF5111 IC 2LH
mAU
250
200
150
100
50
0
10
15
20
25
Volume (mL)
D
Post-FPLC Protein Yields
mg/L
15
10
5
0
IL-2
miniF5111IC 2HL
miniF5111IC 2LH
miniF5111 IC HL2
miniF5111 IC LH2
miniControl IC

Figure S1. Expression and purification of miniF5111 IC variants. (A) SDS-PAGE analysis of purified miniControl IC. **(B)** Size-exclusion chromatography (SEC) profile overlay of miniF5111 IC variants (2HL, 2LH, HL2, and LH2). **(C)** SEC trace of miniF5111 IC (2LH format) purified under optimized conditions. **(D)** Post-FPLC protein yields (mg/L) for each miniF5111 IC variant compared with IL-2 and miniControl IC. Data are representative of two independent protein preparations.

Figure S2.

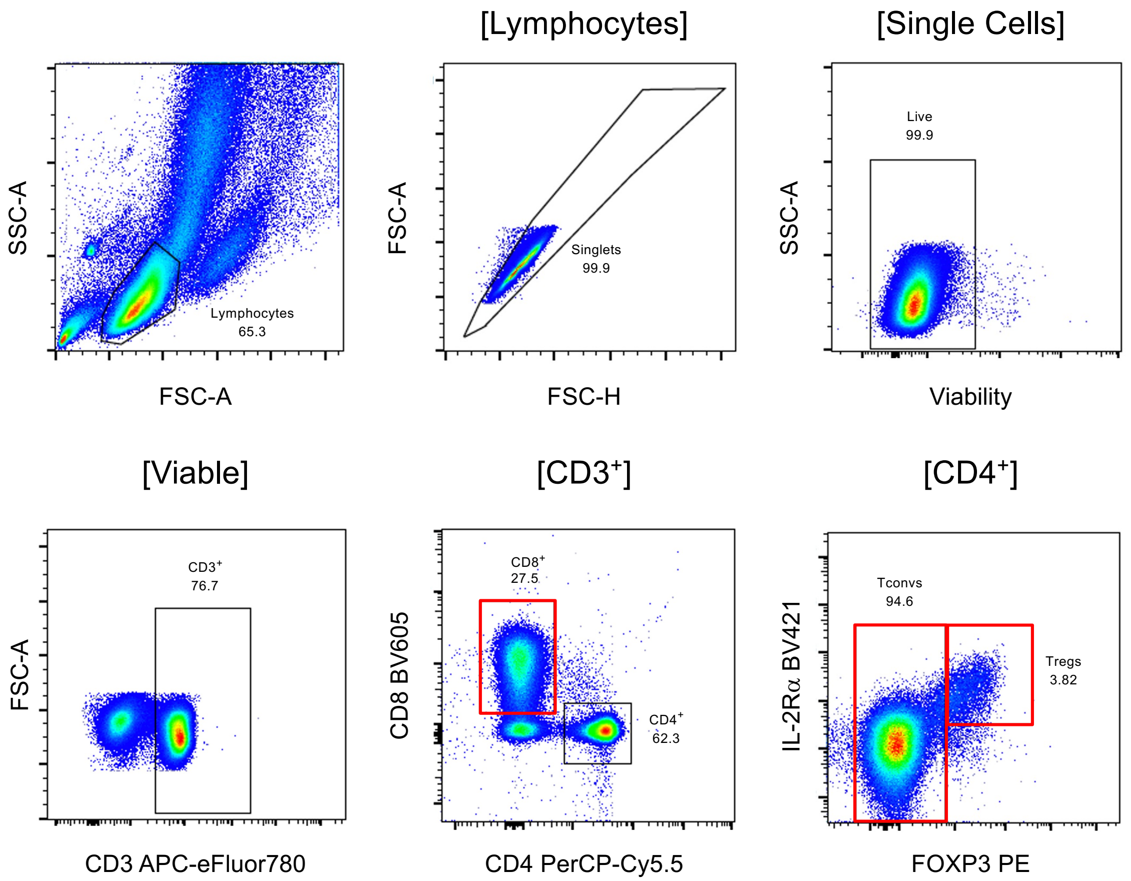
[Lymphocytes]
[Single Cells]
SSC-A
Lymphocytes
65.3
FSC-A
FSC-A
Singlets
99.9
FSC-H
SSC-A
Live
99.9
Viability
[Viable]
[CD3+]
[CD4+]
FSC-A
CD3+
76.7
CD3 APC-eFluor780
CD8 BV605
CD8+
27.5
CD4+
62.3
CD4 PerCP-Cy5.5
IL-2Rα BV421
Tconvs
94.6
Tregs
3.82
FOXP3 PE

Figure S2. Representative flow cytometry plots illustrating the gating strategy used for human PBMC signaling studies. All subsets were derived sequentially from the Lymphocytes > Singlets > Live gate. Within this gate, $CD3^+$ T cells were identified and further divided into $CD4^+$ and $CD8^+$ subsets. Tregs were gated as $CD3^+$ > $CD4^+$ > IL-$2R\alpha^+FOXP3^+$, whereas Tconvs were defined as $CD3^+$ > $CD4^+$ > $FOXP3^-$. $CD8^+$T cells were gated as $CD3^+$ > $CD8^+$.

Figure S3.

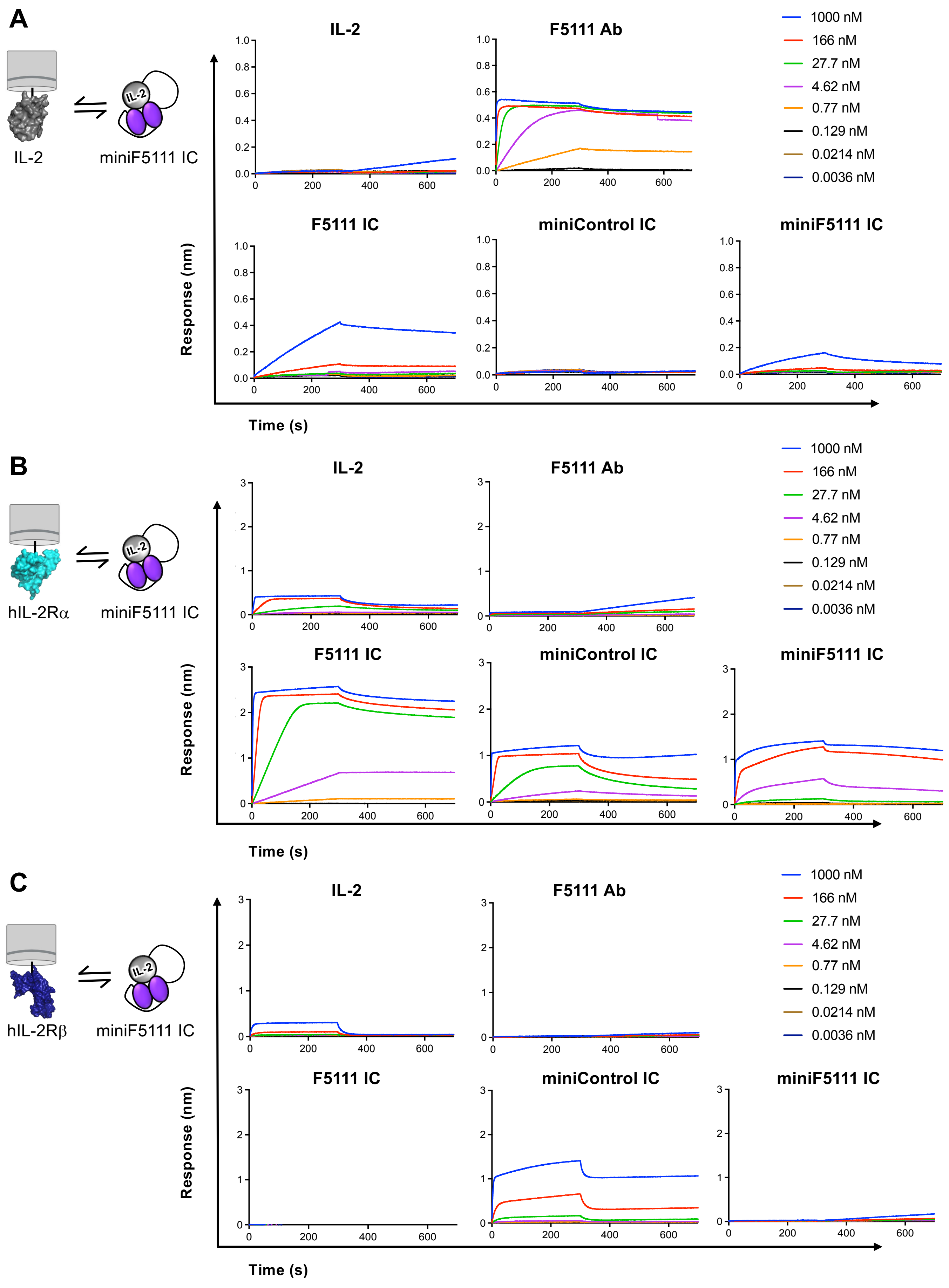
A
IL-2
miniF5111 IC
IL-2
F5111 Ab
F5111 IC
miniControl IC
miniF5111 IC
Response (nm)
Time (s)
1000 nM
166 nM
27.7 nM
4.62 nM
0.77 nM
0.129 nM
0.0214 nM
0.0036 nM
B
hIL-2Rα
miniF5111 IC
IL-2
F5111 Ab
F5111 IC
miniControl IC
miniF5111 IC
Response (nm)
Time (s)
C
hIL-2Rβ
miniF5111 IC
IL-2
F5111 Ab
F5111 IC
miniControl IC
miniF5111 IC
Response (nm)
Time (s)

Figure S3. Interaction of miniF5111 IC with IL-2 and IL-2 receptor subunits. (A-C) Kinetic biolayer interferometry traces depicting the interaction of soluble IL-2, F5111 antibody (Ab), F5111 IC, miniF5111 IC, and miniControl IC with immobilized **(A)** IL-2, **(B)** IL-2Rα, and **(C)** IL-2Rβ.

Figure S4.

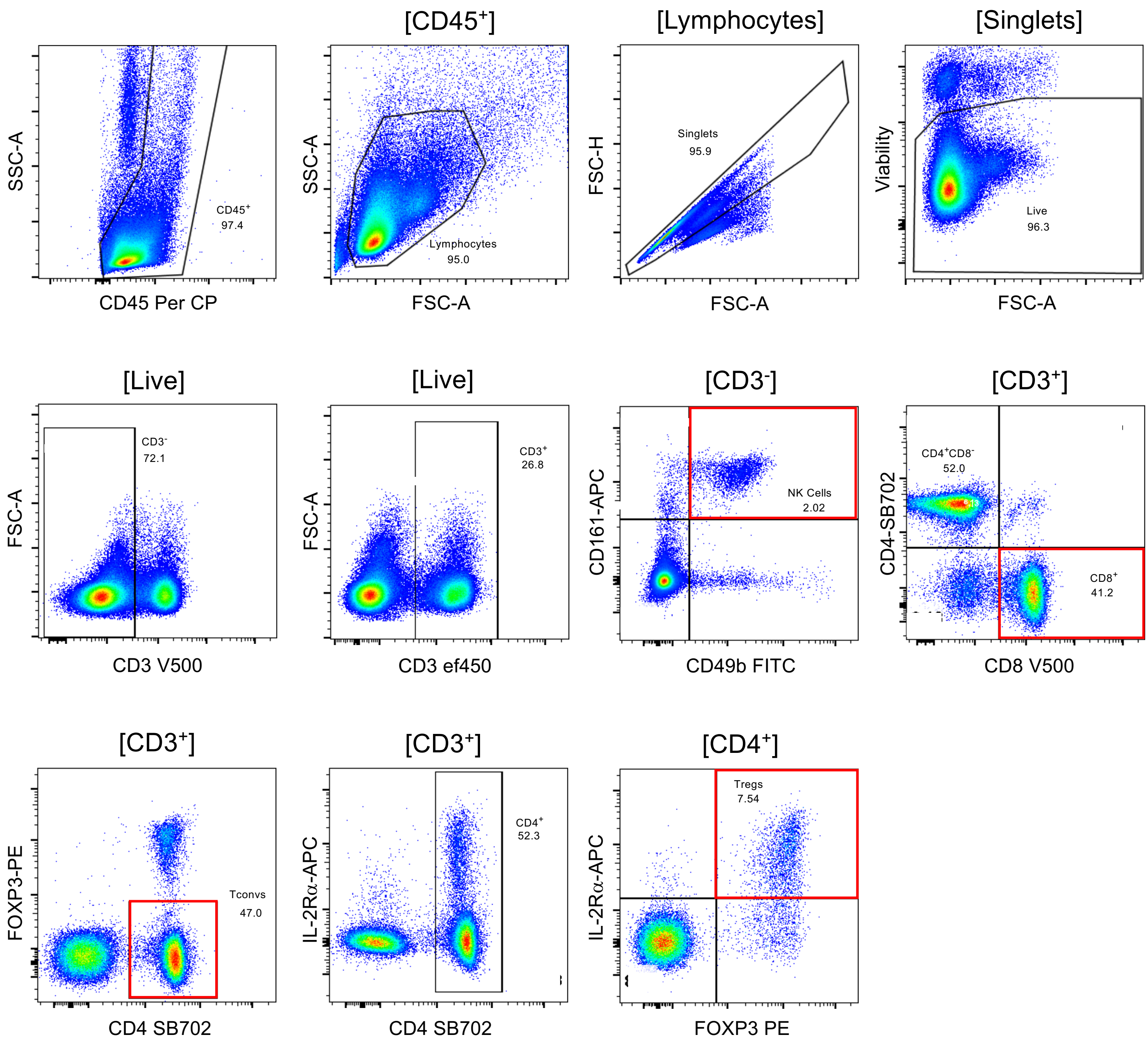

SSC-A
CD45 Per CP
CD45+
97.4
[CD45+]
SSC-A
FSC-A
Lymphocytes
95.0
[Lymphocytes]
FSC-H
FSC-A
Singlets
95.9
[Singlets]
Viability
FSC-A
Live
96.3
[Live]
FSC-A
CD3 V500
CD3-
72.1
[Live]
FSC-A
CD3 ef450
CD3+
26.8
[CD3-]
CD161-APC
CD49b FITC
NK Cells
2.02
[CD3+]
CD4-SB702
CD8 V500
CD4+CD8-
52.0
CD8+
41.2
[CD3+]
FOXP3-PE
CD4 SB702
Tconvs
47.0
[CD3+]
IL-2Rα-APC
CD4 SB702
CD4+
52.3
[CD4+]
IL-2Rα-APC
FOXP3 PE
Tregs
7.54

Figure S4. Representative flow cytometry plots illustrating the gating strategy used for C57BL/6 mouse immune cell subset expansion studies. All subsets were derived sequentially from $CD45^+$ > Lymphocytes > Singlets > Live gate. Within this gate, Tregs were gated as $CD3^+$ > $CD4^+$ > IL-2Rα^+FOXP3$^+$, $CD8^+$ T cells were gated as $CD3^+$ > $CD4^-$ $CD8^+$, Tconvs were gated as $CD3^+$ > $CD4^+FOXP3^-$, and NK cells were gated as $CD3^-$ > $CD161^+CD49b^+$.

Figure S5.

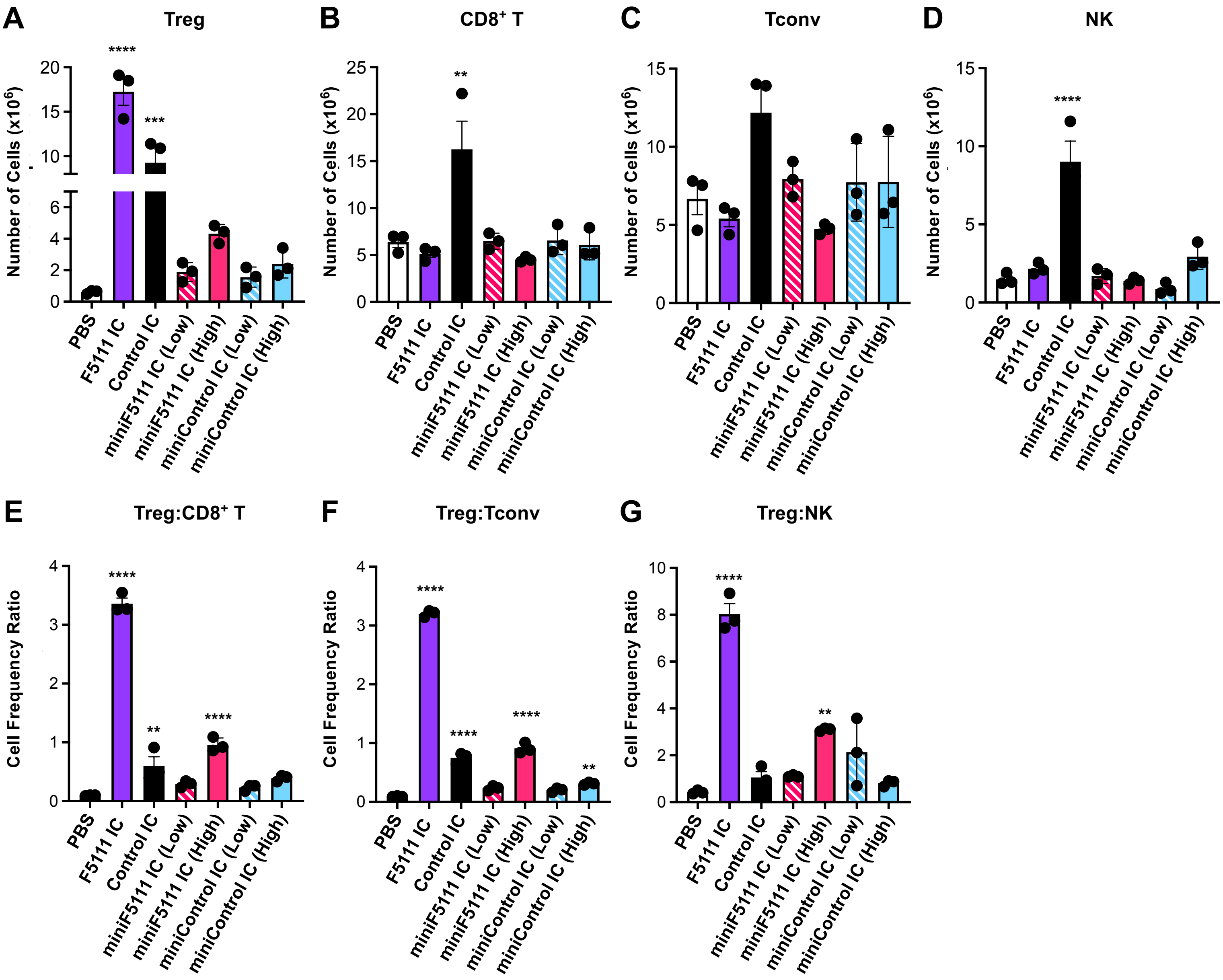

A
Treg
B
CD8⁺ T
C
Tconv
D
NK
E
Treg:CD8⁺ T
F
Treg:Tconv
G
Treg:NK
Number of Cells (x10⁶)
Cell Frequency Ratio
PBS
F5111 IC
Control IC
miniF5111 IC (Low)
miniF5111 IC (High)
miniControl IC (Low)
miniControl IC (High)

Figure S5. In vivo administration of miniF5111 IC demonstrates a dose-dependent expansion of Tregs. (A-G) C57BL/6 mice (n=3 per group) were treated daily for 4 consecutive days with PBS, F5111 IC (1.5 μg IL-2 equivalent), Control IC (1.5 μg IL-2 equivalent), miniF5111 IC (1.5 μg IL-2 equivalent, striped magenta), miniF5111 IC (7.5 μg IL-2 equivalent, solid magenta), miniControl IC (1.5 μg IL-2 equivalent, striped blue), and miniControl IC (7.5 μg IL-2 equivalent, solid blue). Spleens were harvested 24 hours after the last injection for flow cytometry analysis. **(A-D)** Total number of **(A)** Treg, **(B)** $CD8^{+}$T, **(C)** Tconv, and **(D)** NK cells. **(E-G)** Ratios of Treg cells to **(E)** $CD8^{+}$T, **(F)** Tconv, and **(G)** NK cells. Data represent mean ± SD (n=3); Statistical significance was determined by one-way ANOVA with Tukey's multiple comparison test and is noted only for comparisons to PBS. *$p<0.05$, **$p<0.01$, ***$p<0.001$, ****$p<0.0001$. All statistical comparisons are presented in **Supplementary Table S5**.

Figure S6.

A

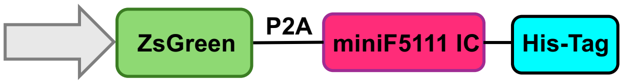
ZsGreen
P2A
miniF5111 IC
His-Tag

B

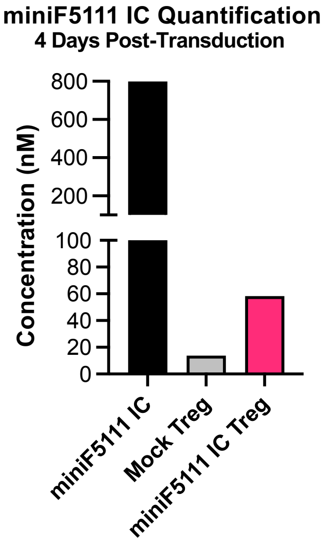
miniF5111 IC Quantification
4 Days Post-Transduction
Concentration (nM)
800
600
400
200
100
80
60
40
20
0
miniF5111 IC
Mock Treg
miniF5111 IC Treg

Figure S6. Retrovirally transduced miniF5111 IC is successfully secreted from Tregs. (A) Schematic of transduced miniF5111 IC construct with P2A-linked ZsGreen reporter. **(B)** Concentration of miniF5111 IC in cell culture supernatant of engineered Tregs 4 days post-transduction, as quantified by ELISA.

Figure S7.

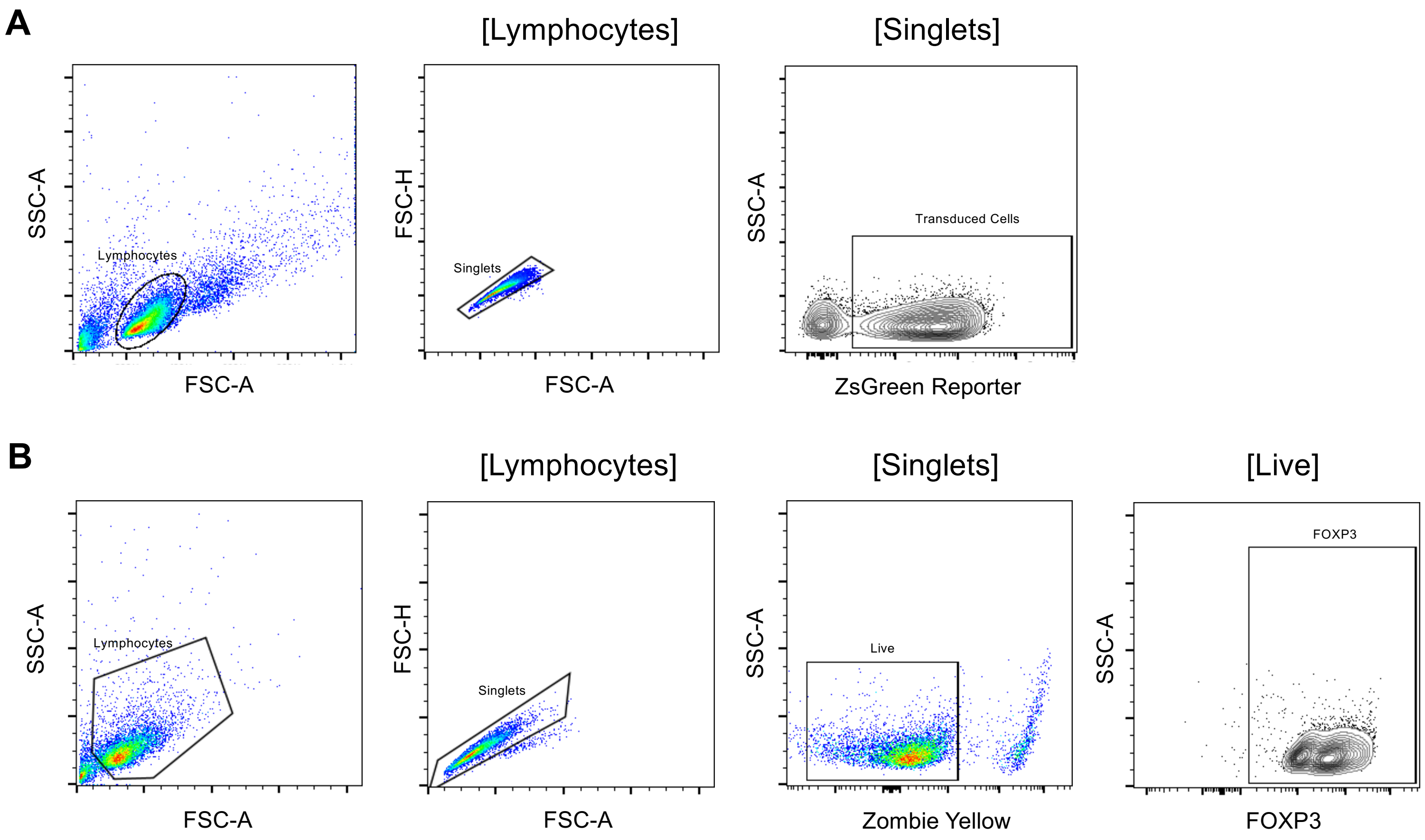
A
[Lymphocytes]
[Singlets]
SSC-A
Lymphocytes
FSC-A
FSC-H
Singlets
FSC-A
SSC-A
Transduced Cells
ZsGreen Reporter
B
[Lymphocytes]
[Singlets]
[Live]
SSC-A
Lymphocytes
FSC-A
FSC-H
Singlets
FSC-A
SSC-A
Live
Zombie Yellow
SSC-A
FOXP3
FOXP3

Figure S7. Representative flow cytometry plots illustrating the gating strategy used for injection day transduction and FOXP3 analysis of Tregs. (A) On the day of adoptive Treg cell transfer, ZsGreen expression was quantified from the Lymphocytes > Singlet gate. **(B)** On the day of adoptive Treg cell transfer, FOXP3 expression was quantified from the Lymphocyte > Singlets > Live cell gate.

Figure S8.

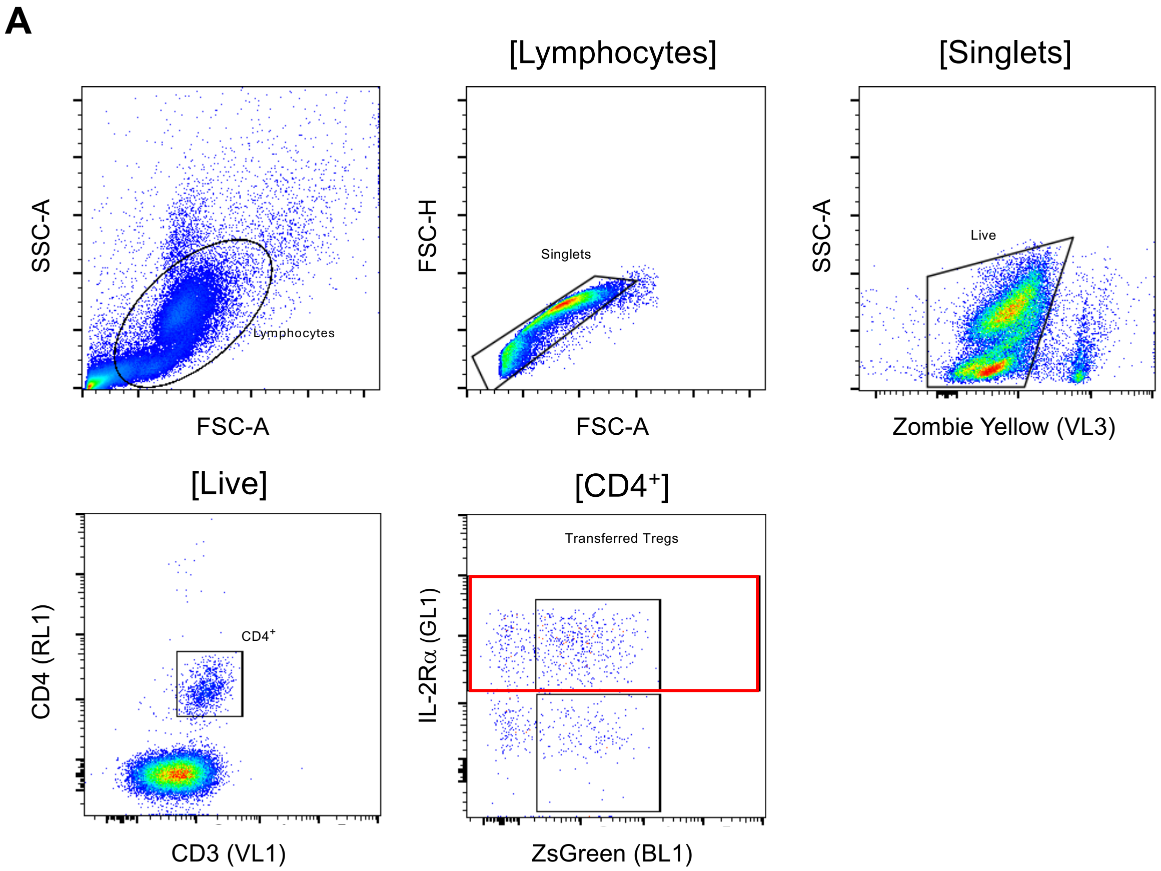
A
[Lymphocytes]
[Singlets]
SSC-A
FSC-A
Lymphocytes
FSC-H
Singlets
Live
Zombie Yellow (VL3)
[Live]
[CD4⁺]
CD4 (RL1)
CD3 (VL1)
CD4⁺
Transferred Tregs
IL-2Rα (GL1)
ZsGreen (BL1)

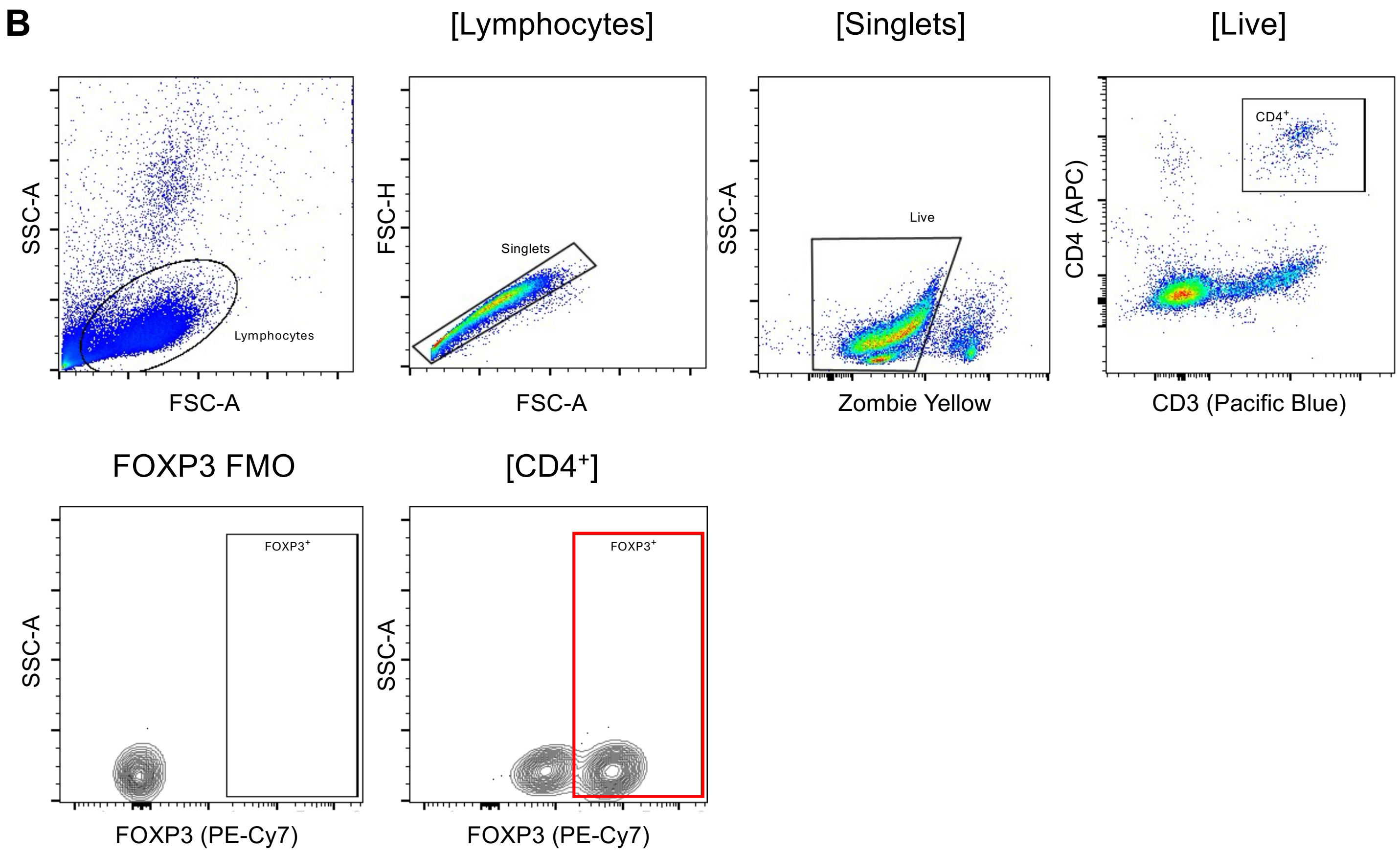
B
[Lymphocytes]
[Singlets]
[Live]
SSC-A
FSC-A
Lymphocytes
FSC-H
Singlets
Live
Zombie Yellow
CD4⁺
CD4 (APC)
CD3 (Pacific Blue)
FOXP3 FMO
[CD4⁺]
FOXP3⁺
FOXP3 (PE-Cy7)

Figure S8. Representative flow cytometry plots illustrating the gating strategy used for engineered Treg adoptive transfer studies. (A) IL-2Rα and ZsGreen expression were quantified from the Lymphocyte > Singlets > Live > $CD3^+$ > $CD4^+$ gate. (**B)** FOXP3 expression was quantified from the Lymphocyte > Singlets > Live > $CD3^+$ $CD4^+$ gate.

Supplementary Table S1. Antibody and IC sequences.

Construct	Amino Acid Sequences<br>Signal sequence ; VH ; VL ; human IgG1 CH1, CH2, and CH3 ; IL-2 ;<br>Linker ; human Lambda CL ; 6xHis tag
miniF5111 IC 2HL	MYRMQLLSCIALSLALVTNSGSAPTSSSTKKTQLQLEHLLLDLQMIL<br>NGINNYKNPKLTRILTFKFYMPKKATELKHLQCLEEELKPLEEVLNLA<br>QSKNFHLRPRDLISNINVIVLELKGSETTFMCEYADETATIVEFLNRW<br>ITFCQSIISTLTGGGGSGGGGSGGGGSGGGGSGGGGSGGGGSG<br>GGGSQLQLQESGPGLVKPSQTLSLTCTVSGGSISSGGYYWSWIRQ<br>HPGKGLEWIGYIYYSGSTYYNPSLKSRVTISVDTSKNQFSLKLSSVT<br>AADTAVYYCARTPTVTGDWFDPWGRGTLVTVSSGGGGSGGGGS<br>GGGGSNFMLTQPHSVSESPGKTVTISCTRSSGSIASNYVQWYQQ<br>RPGSSPTTVIYEDNQRPSGVPDRFSGSIDSSSNSASLTISGLKTED<br>EADYYCQSYDSSNVVFGGGTKLTVLAAAHHHHHH
miniF5111 IC 2LH	MYRMQLLSCIALSLALVTNSGSAPTSSSTKKTQLQLEHLLLDLQMIL<br>NGINNYKNPKLTRMLTFKFYMPKKATELKHLQCLEEELKPLEEVLNL<br>AQSKNFHLRPRDLISNINVIVLELKGSETTFMCEYADETATIVEFLNR<br>WITFCQSIISTLTGGGGSGGGGSGGGGSGGGGSGGGGSGGGGS<br>GGGGSNFMLTQPHSVSESPGKTVTISCTRSSGSIASNYVQWYQQ<br>RPGSSPTTVIYEDNQRPSGVPDRFSGSIDSSSNSASLTISGLKTED<br>EADYYCQSYDSSNVVFGGGTKLTVLGGGGSGGGGSGGGGSQLQ<br>LQESGPGLVKPSQTLSLTCTVSGGSISSGGYYWSWIRQHPGKGLE<br>WIGYIYYSGSTYYNPSLKSRVTISVDTSKNQFSLKLSSVTAADTAVY<br>YCARTPTVTGDWFDPWGRGTLVTVSSAAAHHHHHH
miniF5111 IC HL2	METDTLLLWVLLLWVPGSTGDGSQLQLQESGPGLVKPSQTLSLTC<br>TVSGGSISSGGYYWSWIRQHPGKGLEWIGYIYYSGSTYYNPSLKS<br>RVTISVDTSKNQFSLKLSSVTAADTAVYYCARTPTVTGDWFDPWG<br>RGTLVTVSGGGGSGGGGSGGGGSNFMLTQPHSVSESPGKTVTIS<br>CTRSSGSIASNYVQWYQQRPGSSPTTVIYEDNQRPSGVPDRFSG<br>SIDSSSNSASLTISGLKTEDEADYYCQSYDSSNVVFGGGTKLTVLG<br>GGGSGGGGSGGGGSGGGGSGGGGSGGGGSGGGGSAPTSSST<br>KKTQLQLEHLLLDLQMILNGINNYKNPKLTRMLTFKFYMPKKATELK<br>HLQCLEEELKPLEEVLNLAQSKNFHLRPRDLISNINVIVLELKGSETT<br>FMCEYADETATIVEFLNRWITFCQSIISTLTAAAHHHHHH
miniF5111 IC LH2	MRVPAQLLGLLLLWLPGARCGSNFMLTQPHSVSESPGKTVTISCTR<br>SSGSIASNYVQWYQQRPGSSPTTVIYEDNQRPSGVPDRFSGSIDS<br>SSNSASLTISGLKTEDEADYYCQSYDSSNVVFGGGTKLTVLGGGG<br>SGGGGSGGGGSQLQLQESGPGLVKPSQTLSLTCTVSGGSISSGG<br>YYWSWIRQHPGKGLEWIGYIYYSGSTYYNPSLKSRVTISVDTSKNQ<br>FSLKLSSVTAADTAVYYCARTPTVTGDWFDPWGRGTLVTVSSGGG<br>GSGGGGSGGGGSGGGGSGGGGSGGGGSGGGGSAPTSSSTKKT<br>QLQLEHLLLDLQMILNGINNYKNPKLTRMLTFKFYMPKKATELKHLQ<br>CLEEELKPLEEVLNLAQSKNFHLRPRDLISNINVIVLELKGSETTFMC<br>EYADETATIVEFLNRWITFCQSIISTLTAAAHHHHHH
miniControl IC	MYRMQLLSCIALSLALVTNSGSAPTSSSTKKTQLQLEHLLLDLQMIL<br>NGINNYKNPKLTRMLTFKFYMPKKATELKHLQCLEEELKPLEEVLNL<br>AQSKNFHLRPRDLISNINVIVLELKGSETTFMCEYADETATIVEFLNR<br>WITFCQSIISTLTGGGGSGGGGSGGGGSGGGGSGGGGSGGGGS<br>GGGGSSVLTQPSSVSAAPGQKVTISCSGSTSNIGNNYVSWYQQH<br>PGKAPKLMIYDVSKRPSGVPDRFSGSKSGNSASLDISGLQSEDEA<br>DYYCAAWDDSLSEFLFGTGTKLTVLGGGGGSGGGGSGGGGSQV<br>QLVESGGNLVQPGGSLRLSCAASGFTFGSFSMSWVRQAPGGGLE<br>WVAGLSARSSLTHYADSVKGRFTISRDNAKNSVYLQMNSLRVEDT<br>AVYYCARRSYDSSGYWGHFYSYMDVWGQGTLVTVSAAAHHHHH<br>H

Abbreviations: $\mathbf{V_H}$, variable domain of the antibody heavy chain; $\mathbf{V_L}$, variable domain of the antibody light chain; $\mathbf{C_H}$**1**, $\mathbf{C_H}$**2**, $\mathbf{C_H}$**3**, constant domains 1-3 of the human IgG1 heavy chain; $\mathbf{C_L}$, constant light chain; **6xHis**, hexahistidine affinity tag.

Supplementary Table S1. Antibody and IC sequences.

Construct	**Amino Acid Sequences**<br>Signal sequence ; V_H ; V_L ; human IgG1 C_H1, C_H2, and C_H3 ; IL-2 ;<br>Linker ; human Lambda C_L ; 6xHis tag
F5111 Antibody/IC Heavy Chain (N297A)	METDTLLLWVLLLWVPGSTGDQLQLQESGPGLVKPSQTLSLTCTVS<br>GGSISSGGYYWSWIRQHPGKGLEWIGYIYYSGSTYYNPSLKSRVTI<br>SVDTSKNQFSLKLSSVTAADTAVYYCARTPTVTGDWFDPWGRGTL<br>VTVSSASTKGPSVFPLAPSSKSTSGGTAALGCLVKDYFPEPVTVS<br>WNSGALTSGVHTFPAVLQSSGLYSLSSVVTVPSSSLGTQTYICNVN<br>HKPSNTKVDKKVEPKSCDKTHTCPPCPAPELLGGPSVFLFPPKPK<br>DTLMISRTPEVTCVVVDVSHEDPEVKFNWYVDGVEVHNAKTKPRE<br>EQYASTYRVVSVLTVLHQDWLNGKEYKCKVSNKALPAPIEKTISKA<br>KGQPREPQVYTLPPSREEMTKNQVSLTCLVKGFYPSDIAVEWESN<br>GQPENNYKTTPPVLDSDGSFFLYSKLTVDKSRWQQGNVFSCSVM<br>HEALHNHYTQKSLSLSPGK
F5111 Antibody Light Chain	MRVPAQLLGLLLLWLPGARCGSNFMLTQPHSVSESPGKTVTISCTR<br>SSGSIASNYVQWYQQRPGSSPTTVIYEDNQRPSGVPDRFSGSIDS<br>SSNSASLTISGLKTEDEADYYCQSYDSSNVVFGGGTKLTVLGQPKA<br>APSVTLFPPSSEELQANKATLVCLISDFYPGAVTVAWKADSSPVKA<br>GVETTTPSKQSNNKYAASSYLSLTPEQWKSHRSYSCQVTHEGSTV<br>EKTVAPTECS
F5111 IC Light Chain	MYRMQLLSCIALSLALVTNSAPTSSSTKKTQLQLEHLLLDLQMILNGI<br>NNYKNPKLTRMLTFKFYMPKKATELKHLQCLEEELKPLEEVLNLAQ<br>SKNFHLRPRDLISNINVIVLELKGSETTFMCEYADETATIVEFLNRWI<br>TFCQSIISTLTGGGGSGGGGSGGGGSGGGGSGGGGSGGGGSGG<br>GGSNFMLTQPHSVSESPGKTVTISCTRSSGSIASNYVQWYQQRPG<br>SSPTTVIYEDNQRPSGVPDRFSGSIDSSSNSASLTISGLKTEDEADY<br>YCQSYDSSNVVFGGGTKLTVLGQPKAAPSVTLFPPSSEELQANKA<br>TLVCLISDFYPGAVTVAWKADSSPVKAGVETTTPSKQSNNKYAASS<br>YLSLTPEQWKSHRSYSCQVTHEGSTVEKTVAPTECS
Control IC Heavy Chain (N297A)	METDTLLLWVLLLWVPGSTGDQVQLVESGGNLVQPGGSLRLSCAA<br>SGFTFGSFSMSWVRQAPGGGLEWVAGLSARSSLTHYADSVKGRF<br>TISRDNAKNSVYLQMNSLRVEDTAVYYCARRSYDSSGYWGHFYSY<br>MDVWGQGTLVTVSASTKGPSVFPLAPSSKSTSGGTAALGCLVKDY<br>FPEPVTVSWNSGALTSGVHTFPAVLQSSGLYSLSSVVTVPSSSLGT<br>QTYICNVNHKPSNTKVDKKVEPKSCDKTHTCPPCPAPELLGGPSV<br>FLFPPKPKDTLMISRTPEVTCVVVDVSHEDPEVKFNWYVDGVEVH<br>NAKTKPREEQYASTYRVVSVLTVLHQDWLNGKEYKCKVSNKALPA<br>PIEKTISKAKGQPREPQVYTLPPSREEMTKNQVSLTCLVKGFYPSDI<br>AVEWESNGQPENNYKTTPPVLDSDGSFFLYSKLTVDKSRWQQGN<br>VFSCSVMHEALHNHYTQKSLSLSPGK
Control IC Light Chain	MYRMQLLSCIALSLALVTNSAPTSSSTKKTQLQLEHLLLDLQMILNGI<br>NNYKNPKLTRMLTFKFYMPKKATELKHLQCLEEELKPLEEVLNLAQ<br>SKNFHLRPRDLISNINVIVLELKGSETTFMCEYADETATIVEFLNRWI<br>TFCQSIISTLTGGGGSGGGGSGGGGSGGGGSGGGGSGGGGSGG<br>GGSSVLTQPSSVSAAPGQKVTISCSGSTSNIGNNYVSWYQQHPGK<br>APKLMIYDVSKRPSGVPDRFSGSKSGNSASLDISGLQSEDEADYY<br>CAAWDDSLSEFLFGTGTKLTVLGGQPKAAPSVTLFPPSSEELQAN<br>KATLVCLISDFYPGAVTVAWKADSSPVKAGVETTTPSKQSNNKYAA<br>SSYLSLTPEQWKSHRSYSCQVTHEGSTVEKTVAPTECS

Abbreviations: $\mathbf{V_H}$, variable domain of the antibody heavy chain; $\mathbf{V_L}$, variable domain of the antibody light chain; $\mathbf{C_H1}$, $\mathbf{C_H2}$, $\mathbf{C_H3}$, constant domains 1-3 of the human IgG1 heavy chain; $\mathbf{C_L}$, constant light chain; **6xHis**, hexahistidine affinity tag.

Supplementary Table S2. Signaling parameters on IL-2Rα$^+$ or IL-2Rα$^-$ YT-1 cells and various human PBMC subsets.

Treatment	EC_{50} (nM)					E_{Max} (nM)				
	IL-2Rα$^+$ YT-1	IL-2Rα$^-$ YT-1	CD3$^+$CD4$^+$ CD8$^-$ FOXP3$^+$ PBMC (Tregs)	CD3$^+$CD4$^-$ CD8$^+$ PBMC (CD8$^+$ T)	CD3$^+$CD4$^+$ CD8$^-$ FOXP3$^-$ PBMC (Tconv)	IL-2Rα$^+$ YT-1	IL-2Rα$^-$ YT-1	CD3$^+$CD4$^+$ CD8$^-$ FOXP3$^+$ PBMC (Tregs)	CD3$^+$CD4$^-$ CD8$^+$ PBMC (CD8$^+$ T)	CD3$^+$CD4$^+$ CD8$^-$ FOXP3$^-$ PBMC (Tconv)
IL-2	0.0155	0.727	0.00315	2.21	0.0538	80.3	92.7	1810	1590	965
F5111 IC	0.0667	363	0.275	221	2.35	75.0	94.7	2090	ND	744
miniF5111 IC 2HL	25.3	1400	-	-	-	92.8	ND	-	-	-
miniF5111 IC 2LH	0.0943	203	0.436	482	20.7	80.3	94.0	1820	ND	832
miniF5111 IC LH2	256	5090	-	-	-	ND	ND	-	-	-
miniControl IC	-	-	0.00328	3.93	0.157	-	-	1980	1840	1100

Abbreviations: EC_{50}, half-maximal effective concentration; E_{Max}, maximal response; PBMC, peripheral blood mononuclear cell; ND, not determined

Supplementary Table S3. IL-2 cytokine and receptor binding properties, as measured by biolayer interferometry.

Immobilized	Soluble	Equilibrium	Kinetic Fit Values		
		K_D (nM)	K_D (nM)	k_{on} (1/Ms)	k_{off} (1/s)
IL-2	IL-2	ND	ND	ND	ND
	F5111 Ab	0.958	1.15	5.34×10^{6}	6.15×10^{-3}
	F5111 IC	ND	ND	ND	ND
	miniF5111 IC	ND	ND	ND	ND
	miniControl IC	>2000	ND	ND	ND
IL-2Rα	IL-2	39.1	30.7	1.56×10^{5}	4.77×10^{-3}
	F5111 Ab	ND	ND	ND	ND
	F5111 IC	8.44	0.479	3.08×10^{5}	1.48×10^{-4}
	miniF5111 IC	34.2	33.1	1.51×10^{5}	5.00×10^{-3}
	miniControl IC	14.4	13.5	3.23×10^{5}	4.38×10^{-3}
IL-2Rβ	IL-2	1020	ND	ND	ND
	F5111 Ab	ND	ND	ND	ND
	F5111 IC	ND	ND	ND	ND
	miniF5111 IC	ND	ND	ND	ND
	miniControl IC	275	307	1.04×10^{5}	3.18×10^{-2}

Abbreviations: K_D, equilibrium dissociation constant; k_{on}, association rate constant; k_{off}, dissociation rate constant; Ab, antibody; ND, not determined

Supplementary Table S4. Pharmacokinetic study parameters.

Sample	Slow Half-Life	Fast Half-Life
IL-2	ND	ND
miniF5111 IC	1.56 h	0.128 h
miniControl IC	ND	ND
F5111 IC	52.6 h	1.63 h

Abbreviation: ND, not determined

Supplementary Table S1. Antibody and IC sequences.

Construct	**Amino Acid Sequences**<br>Signal sequence ; V_H ; V_L ; human IgG1 C_H1, C_H2, and C_H3 ; IL-2 ;<br>Linker ; human Lambda C_L ; 6xHis tag
miniF5111 IC 2HL	MYRMQLLSCIALSLALVTNSGSAPTSSSTKKTQLQLEHLLLDLQMIL<br>NGINNYKNPKLTRILTFKFYMPKKATELKHLQCLEEELKPLEEVLNLA<br>QSKNFHLRPRDLISNINVIVLELKGSETTFMCEYADETATIVEFLNRW<br>ITFCQSIISTLTGGGGSGGGGSGGGGSGGGGSGGGGSGGGGSG<br>GGGSQLQLQESGPGLVKPSQTLSLTCTVSGGSISSGGYYWSWIRQ<br>HPGKGLEWIGYIYYSGSTYYNPSLKSRVTISVDTSKNQFSLKLSSVT<br>AADTAVYYCARTPTVTGDWFDPWGRGTLVTVSSGGGGSGGGGS<br>GGGGSNFMLTQPHSVSESPGKTVTISCTRSSGSIASNYVQWYQQ<br>RPGSSPTTVIYEDNQRPSGVPDRFSGSIDSSSNSASLTISGLKTED<br>EADYYCQSYDSSNVVFGGGTKLTVLAAAHHHHHH
miniF5111 IC 2LH	MYRMQLLSCIALSLALVTNSGSAPTSSSTKKTQLQLEHLLLDLQMIL<br>NGINNYKNPKLTRMLTFKFYMPKKATELKHLQCLEEELKPLEEVLNL<br>AQSKNFHLRPRDLISNINVIVLELKGSETTFMCEYADETATIVEFLNR<br>WITFCQSIISTLTGGGGSGGGGSGGGGSGGGGSGGGGSGGGGS<br>GGGGSNFMLTQPHSVSESPGKTVTISCTRSSGSIASNYVQWYQQ<br>RPGSSPTTVIYEDNQRPSGVPDRFSGSIDSSSNSASLTISGLKTED<br>EADYYCQSYDSSNVVFGGGTKLTVLGGGGSGGGGSGGGGSQLQ<br>LQESGPGLVKPSQTLSLTCTVSGGSISSGGYYWSWIRQHPGKGLE<br>WIGYIYYSGSTYYNPSLKSRVTISVDTSKNQFSLKLSSVTAADTAVY<br>YCARTPTVTGDWFDPWGRGTLVTVSSAAAHHHHHH
miniF5111 IC HL2	METDTLLLWVLLLWVPGSTGDGSQLQLQESGPGLVKPSQTLSLTC<br>TVSGGSISSGGYYWSWIRQHPGKGLEWIGYIYYSGSTYYNPSLKS<br>RVTISVDTSKNQFSLKLSSVTAADTAVYYCARTPTVTGDWFDPWG<br>RGTLVTVSGGGGSGGGGSGGGGSNFMLTQPHSVSESPGKTVTIS<br>CTRSSGSIASNYVQWYQQRPGSSPTTVIYEDNQRPSGVPDRFSG<br>SIDSSSNSASLTISGLKTEDEADYYCQSYDSSNVVFGGGTKLTVLG<br>GGGSGGGGSGGGGSGGGGSGGGGSGGGGSGGGGSAPTSSST<br>KKTQLQLEHLLLDLQMILNGINNYKNPKLTRMLTFKFYMPKKATELK<br>HLQCLEEELKPLEEVLNLAQSKNFHLRPRDLISNINVIVLELKGSETT<br>FMCEYADETATIVEFLNRWITFCQSIISTLTAAAHHHHHH
miniF5111 IC LH2	MRVPAQLLGLLLLWLPGARCGSNFMLTQPHSVSESPGKTVTISCTR<br>SSGSIASNYVQWYQQRPGSSPTTVIYEDNQRPSGVPDRFSGSIDS<br>SSNSASLTISGLKTEDEADYYCQSYDSSNVVFGGGTKLTVLGGGG<br>SGGGGSGGGGSQLQLQESGPGLVKPSQTLSLTCTVSGGSISSGG<br>YYWSWIRQHPGKGLEWIGYIYYSGSTYYNPSLKSRVTISVDTSKNQ<br>FSLKLSSVTAADTAVYYCARTPTVTGDWFDPWGRGTLVTVSSGGG<br>GSGGGGSGGGGSGGGGSGGGGSGGGGSGGGGSAPTSSSTKKT<br>QLQLEHLLLDLQMILNGINNYKNPKLTRMLTFKFYMPKKATELKHLQ<br>CLEEELKPLEEVLNLAQSKNFHLRPRDLISNINVIVLELKGSETTFMC<br>EYADETATIVEFLNRWITFCQSIISTLTAAAHHHHHH
miniControl IC	MYRMQLLSCIALSLALVTNSGSAPTSSSTKKTQLQLEHLLLDLQMIL<br>NGINNYKNPKLTRMLTFKFYMPKKATELKHLQCLEEELKPLEEVLNL<br>AQSKNFHLRPRDLISNINVIVLELKGSETTFMCEYADETATIVEFLNR<br>WITFCQSIISTLTGGGGSGGGGSGGGGSGGGGSGGGGSGGGGS<br>GGGGSSVLTQPSSVSAAPGQKVTISCSGSTSNIGNNYVSWYQQH<br>PGKAPKLMIYDVSKRPSGVPDRFSGSKSGNSASLDISGLQSEDEA<br>DYYCAAWDDSLSEFLFGTGTKLTVLGGGGGSGGGGSGGGGSQV<br>QLVESGGNLVQPGGSLRLSCAASGFTFGSFSMSWVRQAPGGGLE<br>WVAGLSARSSLTHYADSVKGRFTISRDNAKNSVYLQMNSLRVEDT<br>AVYYCARRSYDSSGYWGHFYSYMDVWGQGTLVTVSAAAHHHHH<br>H

Abbreviations: V_H, variable domain of the antibody heavy chain; V_L, variable domain of the antibody light chain; C_H1, C_H2, C_H3, constant domains 1-3 of the human IgG1 heavy chain; C_L, constant light chain; **6xHis**, hexahistidine affinity tag.

Supplementary Table S1. Antibody and IC sequences.

Construct	Amino Acid Sequences<br>Signal sequence ; V_H ; V_L ; human IgG1 C_H1, C_H2, and C_H3 ; IL-2 ;<br>Linker ; human Lambda C_L ; 6xHis tag
F5111 Antibody/IC Heavy Chain (N297A)	METDTLLLWVLLLWVPGSTGDQLQLQESGPGLVKPSQTLSLTCTVS<br>GGSISSGGYYWSWIRQHPGKGLEWIGYIYYSGSTYYNPSLKSRVTI<br>SVDTSKNQFSLKLSSVTAADTAVYYCARTPTVTGDWFDPWGRGTL<br>VTVSSASTKGPSVFPLAPSSKSTSGGTAALGCLVKDYFPEPVTVS<br>WNSGALTSGVHTFPAVLQSSGLYSLSSVVTVPSSSLGTQTYICNVN<br>HKPSNTKVDKKVEPKSCDKTHTCPPCPAPELLGGPSVFLFPPKPK<br>DTLMISRTPEVTCVVVDVSHEDPEVKFNWYVDGVEVHNAKTKPRE<br>EQYASTYRVVSVLTVLHQDWLNGKEYKCKVSNKALPAPIEKTISKA<br>KGQPREPQVYTLPPSREEMTKNQVSLTCLVKGFYPSDIAVEWESN<br>GQPENNYKTTPPVLDSDGSFFLYSKLTVDKSRWQQGNVFSCSVM<br>HEALHNHYTQKSLSLSPGK
F5111 Antibody Light Chain	MRVPAQLLGLLLLWLPGARCGSNFMLTQPHSVSESPGKTVTISCTR<br>SSGSIASNYVQWYQQRPGSSPTTVIYEDNQRPSGVPDRFSGSIDS<br>SSNSASLTISGLKTEDEADYYCQSYDSSNVVFGGGTKLTVLGQPKA<br>APSVTLFPPSSEELQANKATLVCLISDFYPGAVTVAWKADSSPVKA<br>GVETTTPSKQSNNKYAASSYLSLTPEQWKSHRSYSCQVTHEGSTV<br>EKTVAPTECS
F5111 IC Light Chain	MYRMQLLSCIALSLALVTNSAPTSSSTKKTQLQLEHLLLDLQMILNGI<br>NNYKNPKLTRMLTFKFYMPKKATELKHLQCLEEELKPLEEVLNLAQ<br>SKNFHLRPRDLISNINVIVLELKGSETTFMCEYADETATIVEFLNRWI<br>TFCQSIISTLTGGGGSGGGGSGGGGSGGGGSGGGGSGGGGSGG<br>GGSNFMLTQPHSVSESPGKTVTISCTRSSGSIASNYVQWYQQRPG<br>SSPTTVIYEDNQRPSGVPDRFSGSIDSSSNSASLTISGLKTEDEADY<br>YCQSYDSSNVVFGGGTKLTVLGQPKAAPSVTLFPPSSEELQANKA<br>TLVCLISDFYPGAVTVAWKADSSPVKAGVETTTPSKQSNNKYAASS<br>YLSLTPEQWKSHRSYSCQVTHEGSTVEKTVAPTECS
Control IC Heavy Chain (N297A)	METDTLLLWVLLLWVPGSTGDQVQLVESGGNLVQPGGSLRLSCAA<br>SGFTFGSFSMSWVRQAPGGGLEWVAGLSARSSLTHYADSVKGRF<br>TISRDNAKNSVYLQMNSLRVEDTAVYYCARRSYDSSGYWGHFYSY<br>MDVWGQGTLVTVSASTKGPSVFPLAPSSKSTSGGTAALGCLVKDY<br>FPEPVTVSWNSGALTSGVHTFPAVLQSSGLYSLSSVVTVPSSSLGT<br>QTYICNVNHKPSNTKVDKKVEPKSCDKTHTCPPCPAPELLGGPSV<br>FLFPPKPKDTLMISRTPEVTCVVVDVSHEDPEVKFNWYVDGVEVH<br>NAKTKPREEQYASTYRVVSVLTVLHQDWLNGKEYKCKVSNKALPA<br>PIEKTISKAKGQPREPQVYTLPPSREEMTKNQVSLTCLVKGFYPSDI<br>AVEWESNGQPENNYKTTPPVLDSDGSFFLYSKLTVDKSRWQQGN<br>VFSCSVMHEALHNHYTQKSLSLSPGK
Control IC Light Chain	MYRMQLLSCIALSLALVTNSAPTSSSTKKTQLQLEHLLLDLQMILNGI<br>NNYKNPKLTRMLTFKFYMPKKATELKHLQCLEEELKPLEEVLNLAQ<br>SKNFHLRPRDLISNINVIVLELKGSETTFMCEYADETATIVEFLNRWI<br>TFCQSIISTLTGGGGSGGGGSGGGGSGGGGSGGGGSGGGGSGG<br>GGSSVLTQPSSVSAAPGQKVTISCSGSTSNIGNNYVSWYQQHPGK<br>APKLMIYDVSKRPSGVPDRFSGSKSGNSASLDISGLQSEDEADYY<br>CAAWDDSLSEFLFGTGTKLTVLGGQPKAAPSVTLFPPSSEELQAN<br>KATLVCLISDFYPGAVTVAWKADSSPVKAGVETTTPSKQSNNKYAA<br>SSYLSLTPEQWKSHRSYSCQVTHEGSTVEKTVAPTECS

Abbreviations: $\mathbf{V_H}$, variable domain of the antibody heavy chain; $\mathbf{V_L}$, variable domain of the antibody light chain; $\mathbf{C_H1}$, $\mathbf{C_H2}$, $\mathbf{C_H3}$, constant domains 1-3 of the human IgG1 heavy chain; $\mathbf{C_L}$, constant light chain; **6xHis**, hexahistidine affinity tag.

Supplementary Table S2. Signaling parameters on IL-2Rα$^+$ or IL-2Rα$^-$ YT-1 cells and various human PBMC subsets.

Treatment	EC_{50} (nM)					E_{Max} (nM)				
	IL-2Rα$^+$ YT-1	IL-2Rα$^-$ YT-1	CD3$^+$CD4$^+$ CD8$^-$ FOXP3$^+$ PBMC (Tregs)	CD3$^+$CD4$^-$ CD8$^+$ PBMC (CD8$^+$ T)	CD3$^+$CD4$^+$ CD8$^-$ FOXP3$^-$ PBMC (Tconv)	IL-2Rα$^+$ YT-1	IL-2Rα$^-$ YT-1	CD3$^+$CD4$^+$ CD8$^-$ FOXP3$^+$ PBMC (Tregs)	CD3$^+$CD4$^-$ CD8$^+$ PBMC (CD8$^+$ T)	CD3$^+$CD4$^+$ CD8$^-$ FOXP3$^-$ PBMC (Tconv)
IL-2	0.0155	0.727	0.00315	2.21	0.0538	80.3	92.7	1810	1590	965
F5111 IC	0.0667	363	0.275	221	2.35	75.0	94.7	2090	ND	744
miniF5111 IC 2HL	25.3	1400	-	-	-	92.8	ND	-	-	-
miniF5111 IC 2LH	0.0943	203	0.436	482	20.7	80.3	94.0	1820	ND	832
miniF5111 IC LH2	256	5090	-	-	-	ND	ND	-	-	-
miniControl IC	-	-	0.00328	3.93	0.157	-	-	1980	1840	1100

Abbreviations: EC_{50}, half-maximal effective concentration; E_{Max}, maximal response; PBMC, peripheral blood mononuclear cell; ND, not determined

Supplementary Table S3. IL-2 cytokine and receptor binding properties, as measured by biolayer interferometry.

Immobilized	Soluble	Equilibrium	Kinetic Fit Values		
		K_D (nM)	K_D (nM)	k_{on} (1/Ms)	k_{off} (1/s)
IL-2	IL-2	ND	ND	ND	ND
	F5111 Ab	0.958	1.15	5.34×10^{6}	6.15×10^{-3}
	F5111 IC	ND	ND	ND	ND
	miniF5111 IC	ND	ND	ND	ND
	miniControl IC	>2000	ND	ND	ND
IL-2Rα	IL-2	39.1	30.7	1.56×10^{5}	4.77×10^{-3}
	F5111 Ab	ND	ND	ND	ND
	F5111 IC	8.44	0.479	3.08×10^{5}	1.48×10^{-4}
	miniF5111 IC	34.2	33.1	1.51×10^{5}	5.00×10^{-3}
	miniControl IC	14.4	13.5	3.23×10^{5}	4.38×10^{-3}
IL-2Rβ	IL-2	1020	ND	ND	ND
	F5111 Ab	ND	ND	ND	ND
	F5111 IC	ND	ND	ND	ND
	miniF5111 IC	ND	ND	ND	ND
	miniControl IC	275	307	1.04×10^{5}	3.18×10^{-2}

Abbreviations: K_D, equilibrium dissociation constant; k_{on}, association rate constant; k_{off}, dissociation rate constant; Ab, antibody; ND, not determined

Supplementary Table S4. Pharmacokinetic study parameters.

Sample	Slow Half-Life	Fast Half-Life
IL-2	ND	ND
miniF5111 IC	1.56 h	0.128 h
miniControl IC	ND	ND
F5111 IC	52.6 h	1.63 h

Abbreviation: ND, not determined

Supplementary Table S5. Statistical analyses (Included as an Excel spreadsheet).
